# Human RPL7 and DDX21 function synergistically with HTLV-1 Gag to overcome genomic RNA structural barriers that block tRNA^Pro^ primer annealing

**DOI:** 10.1093/nar/gkag680

**Published:** 2026-07-14

**Authors:** Yu-Ci Syu, Zixi Long, Karin Musier-Forsyth

**Affiliations:** Molecular, Cellular, and Developmental Biology Graduate Program, Department of Chemistry and Biochemistry, Center for RNA Biology, and Center for Retrovirus Research, Ohio State University, Columbus, OH 43210,United States; Molecular, Cellular, and Developmental Biology Graduate Program, Department of Chemistry and Biochemistry, Center for RNA Biology, and Center for Retrovirus Research, Ohio State University, Columbus, OH 43210,United States; Molecular, Cellular, and Developmental Biology Graduate Program, Department of Chemistry and Biochemistry, Center for RNA Biology, and Center for Retrovirus Research, Ohio State University, Columbus, OH 43210,United States

## Abstract

Human T-cell leukemia virus type 1 (HTLV-1), an oncogenic retrovirus, uses human tRNA^Pro^ to prime reverse transcription. How tRNA^Pro^ is annealed to the primer-binding site (PBS), which is embedded in a stable hairpin structure in the genomic RNA, remains unclear. In contrast to human immunodeficiency virus type 1 (HIV-1) nucleocapsid (NC) protein, which robustly chaperones transfer RNA (tRNA) annealing to the HIV-1 PBS, HTLV-1 NC protein displays very weak chaperone function. Recombinantly-purified HTLV-1 Gag was only slightly more effective at chaperoning the annealing of tRNA^Pro^ to the PBS than NC protein. To identify potential HTLV-1 Gag interacting co-chaperones in cells, we performed affinity tagging/purification–mass spectrometry. Two significant hits, ribosomal protein L7 (RPL7) and DDX21, were validated by reciprocal co-IP studies in cells. Domain mapping revealed that HTLV-1 Gag interacts with RPL7 and DDX21 through the zinc fingers of NC protein in an RNA-independent fashion. Both RPL7 and DDX21 are packaged into virions, and each protein alone was more effective than HTLV-1 Gag at annealing tRNA^Pro^ to the PBS. Further synergistic effects were observed for the Gag/RPL7/DDX21 combination in overcoming structural constraints at the PBS to promote tRNA^Pro^ annealing. The mechanistic insights gained from these studies may be exploited for the development of new therapeutic strategies aimed at targeting HTLV-1 RT.

## Graphical Abstract

**Graphical Abstract:**
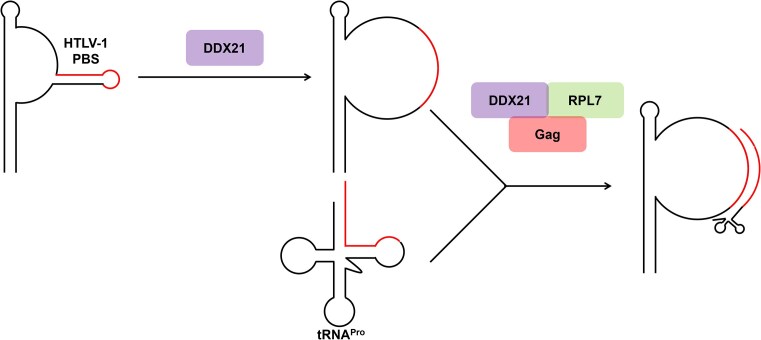

## Introduction

All retroviruses use host cell transfer RNAs (tRNAs) as primers to initiate reverse transcription (RT) [1]. The 3′ 18 nt of the primer tRNA are perfectly complementary to a region in the 5′ untranslated region (UTR) of the genomic RNA (gRNA) known as the primer-binding site (PBS). How the human immunodeficiency virus type 1 (HIV-1) RT primer, tRNA^Lys3^, is selectively packaged into virions and annealed by the nucleocapsid (NC) domain of Gag to the HIV-1 PBS has been extensively studied [2–5]. HIV-1 NC is a robust nucleic acid (NA) chaperone protein [6, 7], displaying duplex destabilization, NA aggregation, and rapid on–off binding kinetics [7–10]. Almost every step in RT, including primer annealing, extension, and multiple strand-transfer steps, requires either Gag or NC’s RNA chaperone activities, which facilitate NA rearrangements and conformational changes [3, 11–13]. The NC domain of HIV-1 Gag is also the major player in gRNA packaging; NC has a preference for binding to single-stranded G residues that are exposed in the packaging signal (Psi) located in the HIV-1 5′ UTR [14]. In contrast, in deltaretroviruses, such as bovine leukemia virus, human T-cell leukemia virus type 1 (HTLV-1), and HTLV-2, matrix (MA) demonstrates stronger RNA chaperone activity and plays a more prominent role in specific Psi RNA binding than NC [15–17]. In our previous study, we found that HTLV-1 uses a specific tRNA^Pro^ isodecoder as the RT primer [18]. However, the viral and/or host factors required for tRNA^Pro^ annealing to the highly-structured HTLV-1 PBS are still unknown.

The HTLV-1 PBS is complementary to the 3′ 18 nt of human tRNA^Pro^. RNA-structure probing data showed that the PBS is embedded in a highly stable hairpin containing 10 Watson–Crick base pairs [17]; this is in contrast to the HIV-1 PBS, which is much less structured [19]. The NC domain of HIV-1 Gag is a robust NA chaperone protein capable of facilitating human tRNA^Lys3^ annealing to the HIV-1 PBS in the absence of any other co-factors [3, 7]. HTLV-1 NC is a relatively weak chaperone protein due to the unique acidic C-terminal extension [7, 20]. We hypothesize that HTLV-1 MA or full-length Gag may be required to facilitate primer tRNA^Pro^ annealing; alternatively, one or more host cell co-factors may be required.

In HIV-1-infected cells, ribosomal protein L7 (RPL7) interacts with the NC domain of HIV-1 Gag and is packaged into HIV-1 virions. RPL7 had a positive synergistic effect on the chaperone activity of HIV-1 Gag; adding both proteins together increased annealing of tRNA^Lys3^ to the HIV-1 PBS [21, 22]. Another host factor, RNA helicase A (RHA/DHX9), also plays a role in HIV-1 RT. RHA interacts with HIV-1 Gag and is packaged into virions [23]; together these proteins change the structure of the HIV-1 5′ UTR to facilitate tRNA^Lys3^ annealing to the PBS [24]. RHA also increases the elongation processivity of HIV-1 reverse transcriptase [25].

In this work, we investigated both viral and host cell factors as potential chaperone proteins capable of facilitating primer annealing to the highly structured HTLV-1 PBS. We successfully expressed and purified recombinant HTLV-1 Gag from *Escherichia coli* (*E. coli*) for the first time and characterized the HTLV-1 Gag interactome in human cells. We identified HTLV-1 Gag-interacting partners, RPL7 and nucleolar RNA helicase 2 (DDX21), which are known chaperone or helicase proteins, respectively, and investigated their roles in regulating RT. Their interactions with HTLV-1 Gag were mapped in cells, and their ability to facilitate tRNA^Pro^ annealing to the PBS was examined *in vitro*. These data suggest that HTLV-1 Gag recruits one or more cellular co-factors to overcome RNA structural barriers and ensure primer placement onto the PBS prior to initiation of RT. Disrupting these direct Gag/co-factor interactions may serve as a new anti-HTLV-1 therapeutic strategy.

## Materials and methods

### Plasmid construction

**Figure 1.**
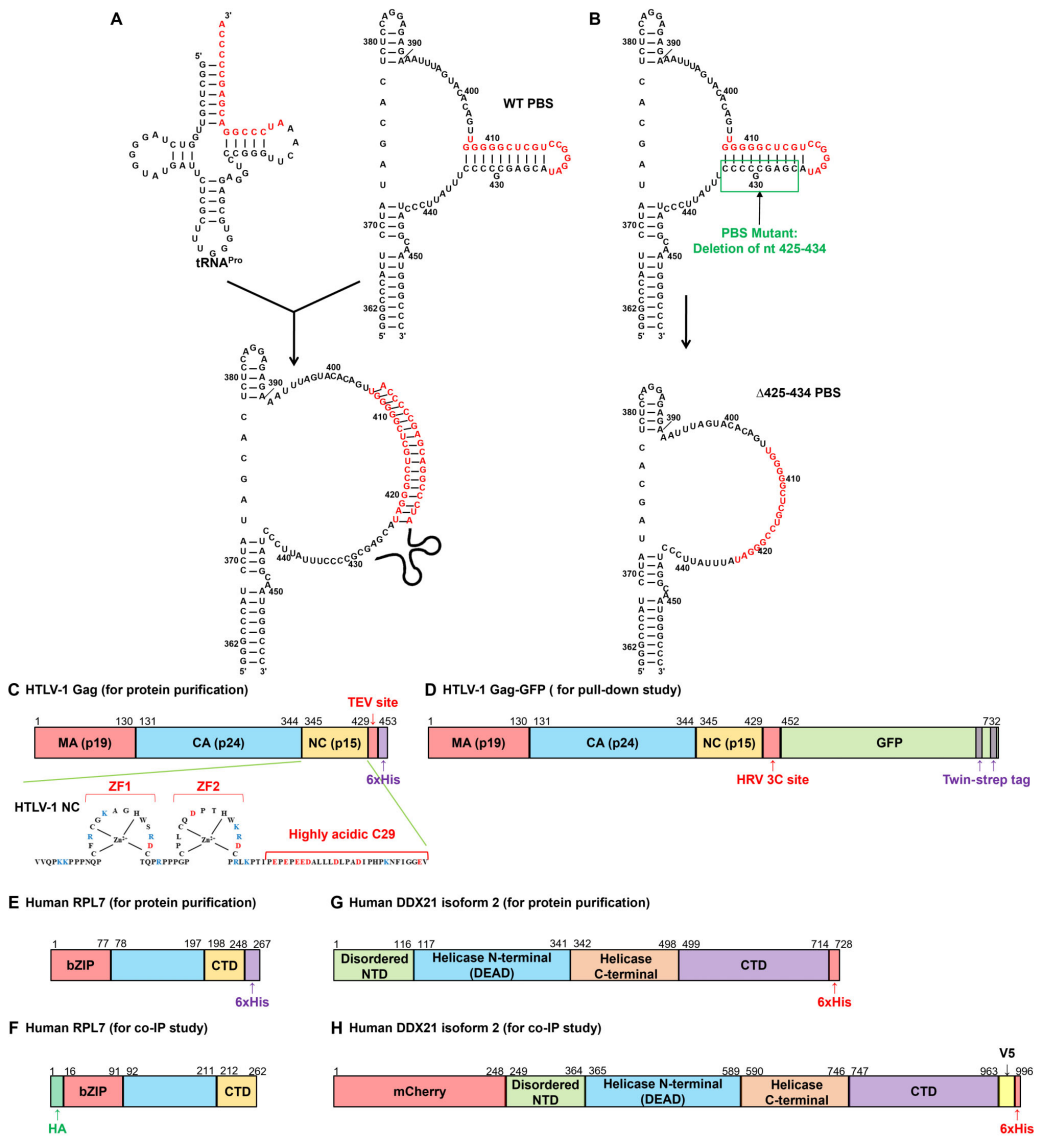
Schematic representation of RNAs and proteins used in this study. (**A**) Sequences and secondary structures of tRNA^Pro^ (75 nt, top left) and a portion of HTLV-1 5′ UTR containing the PBS (98 nt, wild-type (WT) PBS, top right) used in this work. The sequence in the HTLV-1 PBS complementary to the 3′ 18-nt of tRNA^Pro^ is indicated by red letters. The bottom of this panel shows the tRNA^Pro^–PBS annealed complex. The structure of the PBS region is based on the structure-probing results reported in Wu *et al*. [17]. (**B**) Sequence and secondary structure of HTLV-1 PBS region before (WT PBS, top panel) and after deleting nt 425–434 (88 nt, ∆452–434 PBS mutant, bottom of this panel). (**C**) Domain architecture of HTLV-1 Gag bacterial expression construct consisting of MA (p19), capsid (CA, p24), and NC (p17) domains. The Tobacco Etch Virus (TEV) protease cleavage site and a 6×His-tag at the C-terminus are indicated. The sequence of amino acids in the NC domain is shown. (**D**) Domain organization of HTLV-1 Gag mammalian expression construct used in affinity tagging/purification–mass spectrometry (AP-MS) pull-down study; the human rhinovirus (HRV) 3C protease cleavage site, green fluorescent protein (GFP), and Twin-Strep tag at the C-terminus are indicated. (**E**) Domain organization of human RPL7 bacterial expression construct with a 6×His-tag at the C-terminus, an N-terminal basic leucine zipper (bZIP), and a C-terminal domain (CTD). (**F**) Domain organization of human RPL7 mammalian expression construct with hemagglutinin (HA) tag at the N-terminus. (**G**) Domain organization of human DDX21 bacterial expression construct with a 6×His-tag at the C-terminus, a disordered N-terminal domain (NTD), a helicase core (the helicase N-terminal and CTDs), and a CTD. (**H**) Human DDX21 mammalian expression construct with mCherry at the N-terminus and V5 and 6×His tags at the C-terminus.

Plasmid templates for *in vitro* transcribing a portion (nt 362–456) of the HTLV-1 5′ UTR including the PBS region (pUC19 HTLV-1 PBS) and human tRNA^Pro^ (pUC119 tRNA^Pro^) were described previously (Fig. 1A) [18, 26]. To make the HTLV-1 PBS region less structured, nt 425–434 were deleted in pUC19 HTLV-1 PBS using site-directed ligase-independent mutagenesis (Fig. 1B and Supplementary Table S1) [27]. The mutant construct was named pUC19 HTLV-1 ∆425–434 PBS.

To prepare a bacterial expression plasmid for purifying HTLV-1 Gag, the coding sequence of HTLV-1 Gag from N3 HTLV-1 Gag (a gift from Dr Louis Mansky, University of Minnesota) was cloned into the backbone of pET3xc HIV-1 Gag (full-length with p6 domain, a gift from Dr Alan Rein, National Cancer Institute). This plasmid, pET3xc HTLV-1 Gag, contains, from the N-terminus, coding regions for HTLV-1 Gag, a TEV cleavage site (ENLYFQG), and a 6×His-tag (Fig. 1C). To build a mammalian expression construct for HTLV-1 Gag, the coding sequence of HTLV-1 Gag was cut by NheI and BamHI from N3 HTLV-1 Gag and inserted between NheI and BamHI sites in pUCOgs v2.0-1 (a gift from Dr Eddy Arnold, Rutgers University). This construct, pUCOgs HTLV-1 Gag-GFP, contains, from the N-terminus, coding regions for HTLV-1 Gag, HRV 3C protease cleavage site (LEVLFQGP), GFP, and Twin-Strep tag (Fig. 1D). The plasmid for C-terminal FLAG-tagged HTLV-1 Gag (pUCOgs HTLV-1 Gag-FLAG) is derived from pUCOgs HTLV-1 Gag-GFP by swapping HRV 3C site-GFP-Twin-Strep tag with FLAG tag (DYKDDDDK) using NEBuilder HiFi DNA Assembly Kit (New England Biolabs) according to the manufacturer’s instructions. C-terminal 6×His-tagged human RPL7 bacterial expression plasmid, pPB RPL7 (Fig. 1E), was purchased from Applied Biological Materials Inc. N-terminal HA-tagged (YPYDVPDYA) human RPL7 mammalian expression vector, pCMV3 HA-RPL7 (Fig. 1F), was purchased from Sino Biological. Bacterial expression plasmid for purifying human DDX21, pMBPDDX21 (Fig. 1G), was a gift from Dr James Williamson at Scripps Research Institute [28]. N-terminal mCherry and C-terminal V5-tagged DDX21 mammalian expression vector, pLV CMV mCherry-DDX21-V5 (Fig. 1H), was a gift from Dr Larry Gerace at Scripps Research Institute (Addgene plasmid no.175163) [28]. All constructs for mutants of HTLV-1 Gag, RPL7, and DDX21 were also established by NEBuilder HiFi DNA Assembly Kit (Supplementary Table S1).

### Protein expression and purification

C-terminal 6×His-tagged WT and ∆C29 HTLV-1 Gag were expressed in *E. coli* Rosetta (DE3). The culture was grown in autoinduction medium (1% tryptone, 0.5% yeast extract, 0.5% glycerol, 0.05% glucose, 0.2% α-lactose, 25 mM Na_2_HPO_4_, 25 mM KH_2_PO_4_, 50 mM NH_4_Cl, 5 mM Na_2_SO_4_, and 2 mM MgSO_4_) from an OD_600_ of 0.05 to 0.6 at 37°C, and then the protein of interest was auto-induced at 18°C for 28 h. Cell pellets were lyzed by sonication in lysis buffer [(20 mM Tris–HCl, pH 7.4, 1 M NaCl, 1 μM ZnCl_2_, 10 mM 2-mercaptoethanol (β-ME), 10% glycerol, 0.05% Triton X-100, and tablets of protease inhibitor (Roche)]. Polyethylenimine (PEI) was added to the soluble fraction to a final concentration of 0.6% (v/v) to precipitate and remove NAs, and ammonium sulfate was then added to the supernatant to a final concentration of 1.3 M. Ammonium sulfate-containing pellets were resuspended in the buffer A (20 mM Tris–HCl, pH 7.4, 500 mM NaCl, 1 μM ZnCl_2_, and 5 mM β-ME) and then applied to a HIS-Select nickel affinity column (Sigma–Aldrich). The column was washed with buffer A supplemented with 5 mM imidazole and eluted with a step gradient of 10, 20, 50, 75, 100, 150, and 200 mM imidazole. All fractions were analyzed by sodium dodecyl sulfate–polyacrylamide gel electrophoresis (SDS–PAGE) followed by Coomassie blue staining. The elution fractions containing the most concentrated protein of interest were pooled and dialyzed into buffer A to remove imidazole at 4°C overnight. The 6×His tag was cleaved using 3 mg of TEV protease per 20 mg of HTLV-1 Gag during the dialysis. After overnight dialysis and TEV cleavage, the sample was loaded onto a HIS-Select nickel affinity column (Sigma–Aldrich) again to remove the cleaved 6×His tag and 6×His-tagged TEV protease. The NaCl concentration of the fractions containing the purest protein of interest was reduced from 500 to 100 mM by five-fold dilution with the buffer B (20 mM Tris–HCl, pH 7.4, 1 μM ZnCl_2_, and 5 mM β-ME) and applied to a 5 ml HiTrap heparin column (Cytiva). The column was washed with three column volumes (CV) of buffer B with 0.5 M NaCl and eluted with four CV of buffer B with 1 M NaCl. The fractions containing the purest HTLV-1 Gag were combined and dialyzed into buffer A supplemented with 10% glycerol. The protein concentration of HTLV-1 Gag was determined by measuring the absorbance at 280 nm and using a molar extinction coefficient of 60 390 M^−1^cm^−1^.

C-terminal 6×His-tagged human RPL7 was expressed in *E. coli* Rosetta (DE3). The culture was grown in Luria-Bertani (LB) broth (1% tryptone, 0.5% yeast extract, and 0.5% NaCl) from an OD_600_ of 0.05 to 0.6 at 37°C, and the protein of interest was induced with 1 mM of isopropyl β-D-1-thiogalactopyranoside (IPTG) at 37°C for 24 h. The protein was purified as previously described with some modifications [29]. Briefly, cell pellets were dissolved in the denaturing lysis buffer [25 mM Tris–HCl, pH 8.0, 100 mM NaCl, 6 M guanidine hydrochloride, 0.5 mM phenylmethylsulfonyl fluoride (PMSF), and tablets of protease inhibitor (Roche)] to release RPL7 protein from inclusion bodies. The soluble fraction was loaded onto a HIS-Select nickel column (Sigma–Aldrich). The column was washed with 8 CV of denaturing lysis buffer and 8 CV of urea buffer (25 mM Tris–HCl, pH 8.0, 100 mM NaCl, 8 M urea, 10 mM imidazole, and 0.5 mM PMSF) and eluted with 15 CV of urea buffer supplemented with 100 mM of imidazole. The elution fractions containing the purest RPL7 were pooled and dialyzed into 25 mM Tris–HCl, pH 8.0, 150 mM NaCl, 5 mM β-ME, and 10% glycerol. The protein concentrations of RPL7 WT and truncation mutants were examined by measuring the absorbance at 280 nm and using the following molar extinction coefficients: WT, 33 350 M^−1^cm^−1^; ∆bZIP (∆N77), 27 390 M^−1^cm^−1^; ∆CTD (∆C51) 27 850 M^−1^cm^−1^; ∆bZIP/CTD, 21 890 M^−1^cm^−1^.

Human DDX21 with an appended N-terminal maltose-binding protein (MBP) and C-terminal 6×His tag was expressed in *E. coli* Rosetta (DE3). The culture was grown in LB broth from an OD_600_ of 0.05 to 0.6 at 37°C, and then the protein of interest was induced with 1 mM of IPTG at 18°C for 20 h. The protein was purified as previously established protocol with some alterations [28]. Briefly, cells were lyzed by sonication in lysis buffer [20 mM Tris–HCl, pH 7.8, 500 mM NaCl, 1 mM ethylenediaminetetraacetic acid (EDTA), and 1 mM Tris(2-carboxyethyl)phosphine (TCEP), 10% glycerol, 0.05% Triton X-100, and tablets of protease inhibitor (Roche)]. PEI was added to the soluble fraction to a final concentration of 0.025% (v/v) to precipitate and remove NAs, and the clear supernatant was applied to a HIS-Select nickel affinity column (Sigma–Aldrich). The column was washed with buffer A (20 mM Tris–HCl, pH 7.8, 500 mM NaCl, 1 mM EDTA, 1 mM TCEP, and 2% glycerol) supplemented with 5 mM imidazole and eluted with a step gradient of 10, 20, 50, 75, 100, 150, and 200 mM imidazole. The elution fractions containing the most concentrated protein of interest were pooled and dialyzed into buffer A to remove imidazole at 4°C overnight. The MBP tag was cleaved using 3 mg of TEV protease per 20 mg of DDX21 during dialysis. After overnight dialysis and TEV cleavage, the NaCl concentration of the sample was reduced from 500 to 75 mM by 6.7-fold dilution with the buffer B (20 mM Tris–HCl, pH 7.8, 1 mM EDTA, 1 mM TCEP, and 2% glycerol) and loaded onto a 5 ml HiTrap heparin column (Cytiva). The column was washed with three CV of buffer B with 0.5 M NaCl and eluted with four CV of buffer B with 1 M NaCl and 3 CV of buffer B with 2 M NaCl. The fractions containing the purest DDX21 were combined and dialyzed into 20 mM Tris–HCl, pH 7.8, 500 mM NaCl, 1 mM TCEP, and 10% glycerol. The protein concentration of DDX21 was determined from the absorbance at 280 nm and using a molar extinction coefficient of 48 360 M^−1^cm^−1^.

### RNA preparation

Plasmids encoding the HTLV-1 PBS region and human ${\mathrm{tRNA}}_{{\mathrm{UGG}}}^{{\mathrm{Pro}}}$ driven by a T7 promoter with a PstI or FokI site at the 3′ end were linearized and used as a template for *in vitro* transcription using T7 RNA polymerase as previously described (Fig. 1A and B; Supplementary Table S2) [30]. *In vitro*-transcribed RNA was loaded onto a 10% polyacrylamide/8 M urea gel, and the gel piece containing the RNA of interest was excised and eluted in RNA elution buffer (0.5 mM NH_4_OAc and 1 mM EDTA, pH 8.0) at 37°C overnight. The gel eluate was concentrated by butanol extraction and ethanol precipitation. The concentration of RNAs was calculated according to Beer’s law by measuring the absorbance at 260 nm and using the following molar extinction coefficients: HTLV-1 WT PBS, 870 278 M^−1^cm^−1^; HTLV-1 ∆425–434 PBS, 776 828 M^−1^cm^−1^; ${\mathrm{tRNA}}_{{\mathrm{UGG}}}^{{\mathrm{Pro}}}$, 655 343 M^−1^cm^−1^.

Prior to use, all RNAs were folded in 50 mM 4-(2-hydroxyethyl)-1-piperazineethanesulfonic acid (HEPES), pH 7.5 at 80°C for 2 min, 60°C for 2 min, addition of MgCl_2_ to 1 mM, incubation at 37°C for 30 min, and cooling on ice for at least 30 min.

### 5′ ^32^P RNA labeling

To remove the 5′ phosphate before labeling, 500 pmol of *in vitro*-transcribed RNAs was incubated with five units of calf intestinal alkaline phosphatase (New England Biolabs) at 37°C for 1 h. After phenol-chloroform extraction and ethanol precipitation, RNAs were treated with 50 μCi of [γ-^32^P] ATP (PerkinElmer) and 10 units of T4 polynucleotide kinase (New England Biolabs) to phosphorylate the 5′ end. Free [γ-^32^P] ATP was cleaned up by a sephadex G-25 spin column (Roche), and labeled RNAs were recovered by phenol–chloroform extraction and ethanol precipitation.

### Gel-shift tRNA annealing assays

HTLV-1 WT (98 nt) or ∆425–434 PBS (88 nt) and 5′ ^32^P-labeled ${\mathrm{tRNA}}_{{\mathrm{UGG}}}^{{\mathrm{Pro}}}$ (75 nt) were folded separately as described in RNA preparation. Folded HTLV-1 PBS and ${\mathrm{tRNA}}_{{\mathrm{UGG}}}^{{\mathrm{Pro}}}$ were then incubated in the same reaction at 37°C for 10 min and cooled to room temperature before the addition of chaperone proteins (Fig. 1A). After adding chaperone proteins, such as HTLV-1 Gag, human RPL7, and/or human DDX21, the final reaction mixture (10 μl) contained 20 nM 5′ ^32^P-labeled ${\mathrm{tRNA}}_{{\mathrm{UGG}}}^{{\mathrm{Pro}}}$, 200 nM HTLV-1 PBS, 50 mM HEPES, pH 7.5, 150 mM NaCl, 1 mM MgCl_2_, 0.1 mM ZnCl_2_, 5 mM dithiothreitol, and 0–4 μM chaperone proteins. In single-time-point concentration-dependence assays, the reaction mixtures containing different protein concentrations were incubated at 37°C for 1 h. In time-course kinetic assays, the reaction aliquots with a single protein concentration, 0.8 μM RPL7, 0.8 μM DDX21, and/or 2 μM HTLV-1 Gag, were taken at the indicated time point until 1 h at 37°C. For heat annealing (the positive control), HTLV-1 PBS and ${\mathrm{tRNA}}_{{\mathrm{UGG}}}^{{\mathrm{Pro}}}$ were folded together as described in RNA preparation. In all annealing assays, reactions were quenched with 1% SDS and 1 mg/ml proteinase K (New England Biolabs) and incubated at 37°C for 30 min. After phenol-chloroform extraction, the samples were mixed with 6× native loading dye (50% glycerol, 0.25% bromophenol blue, 0.25% xylene cyanol) and loaded onto pre-cast NativePAGE Bis-Tris 4%–16% polyacrylamide gradient gels (Invitrogen). The running buffer was 1× TB (89 mM Tris–HCl and 89 mM boric acid, pH 8.3) supplemented with 1 mM MgCl_2_. Gels were exposed to phosphor screens overnight, and bands were visualized with Typhoon FLA 9500 (Cytiva) and quantified using ImageJ software. Annealed (%) was calculated based on quantifying the intensity of bands as follows: Annealed (%) = [(B1 + B2) / (Free + B1 + B2)] × 100. Time-course annealing data were fit to the following single-exponential equation using GraphPad Prism software: Annealed(t) = Annealed_max_ – (Annealed_max _− Annealed_min_) × e^−kt^, where Annealed (t) is the fraction ${\mathrm{tRNA}}_{{\mathrm{UGG}}}^{{\mathrm{Pro}}}$ annealed as a function of time and k is the annealing rate. Concentration-dependence annealing data were fit to the following equation: Annealed (C) = (Annealed_max_ × *C*) / (*K*_1/2 _+ *C*), where Annealed (*C*) is the fraction ${\mathrm{tRNA}}_{{\mathrm{UGG}}}^{{\mathrm{Pro}}}$ annealed as a function of protein concentration and *K*_1/2_ is the protein concentration when the annealing is half maximal.

### Cell culture and virus fractionation

MT-2 cells were cultured in Roswell Park Memorial Institute 1640 medium supplemented with 10% (v/v) fetal bovine serum (FBS), 100 unit/ml penicillin, and 100 μg/ml streptomycin. Human embryonic kidney cells (HEK293T) were grown in Dulbecco’s Modified Eagle Medium supplemented with 10% FBS, 100 unit/ml penicillin, 100 μg/ml streptomycin, and 1× non-essential amino acids solution. All cells are maintained in a 37°C incubator with 5% CO_2_.

To collect HTLV-1 virions, 10 ml of culture supernatant from MT-2 cells was filtered through 0.45-μm syringe filters, layered on the top of 1 ml of 25% sucrose, and concentrated by ultracentrifugation at 90 000 × *g* for 1.5 h at 4°C in a Sorvall SW41 swinging bucket rotor (sucrose cushion). The viral pellet was resuspended in 500 μl of 1× STE buffer (10 mM Tris–HCl, pH 8.0, 100 mM NaCl, and 1 mM EDTA) by gentle shaking at 4°C overnight. Resuspended viruses were applied to 10 ml of OptiPrep density gradient medium (iodixanol, Sigma–Aldrich) as five steps with 7.5% increments ranging from 10% to 40% and centrifuged at 250 000 × *g* for 3 h at 4°C in a SW41 rotor. After ultracentrifugation, the sample was divided equally from the top to the bottom into nine fractions. These nine fractions were concentrated using sucrose cushion as described earlier and analyzed by immunoblotting.

### Affinity tagging/purification–mass spectrometry

One 100-mm dish seeded with 4 million HEK293T cells were transfected with 10 μg of plasmid expressing GFP or HTLV-1 Gag-GFP (Fig. 1D) by PEI method next day after seeding and harvested 48 h post-transfection [31]. Thirty million cells harvested from three 100-mm dishes were lysed in 1 ml of lysis buffer [100 mM HEPES, pH 8.0, 150 mM NaCl, 0.5% 3-((3-cholamidopropyl) dimethylammonio)-1-propanesulfonate (CHAPS), 1 mM TCEP, 25 mM NaF, and tablets of protease inhibitor (Roche)] with gentle rocking at 4°C for 30 min. Crude cell lysate was cleared of debris via centrifugation at 15 000 × *g* at 4°C for 30 min. MegStrep XT type3 beads (5 μl, IBA Lifesciences) were added to the clear cell lysate, and samples were rotated at 4°C overnight. Beads were washed with 1 ml of 100 mM HEPES, pH 8.0, 150 mM NaCl, and 1 mM TCEP three times and resuspended in 20 μl of phosphate-buffered saline (PBS, 2.67 mM KCl, 137.93 mM NaCl, 1.47 mM KH_2_PO_4_, 8.06 mM Na_2_HPO_4_, pH 7, Gibco). One-fourth of the sample was analyzed by SDS–PAGE followed by Coomassie blue staining to check whether our protein of interest was pulled down (Supplementary Fig. S3). The rest of the sample was further processed by in-bead trypsin proteolysis and analyzed by liquid chromatography with tandem mass spectrometry (LC-MS/MS) peptide sequencing at the Mass Spectrometry and Proteomics (MSP) facility, Campus Chemical Instrument Center (CCIC), Ohio State University.

### Immunoprecipitation (IP) and immunoblotting

HEK293T cells transfected with indicated plasmids by PEI method were harvested 48 h post-transfection. Ten million cells were lysed in 0.5 ml of lysis buffer [PBS supplemented with 1% Triton X-100 and tablets of protease inhibitor (Roche)] with gentle rotating at 4°C for 30 min. Crude cell lysate was centrifuged at 15 000 × g at 4°C for 30 min to remove cell debris. The concentration of protein in the clear cell lysate was determined by Pierce bicinchoninic acid protein assay kit (Thermo Scientific). For HTLV-1 Gag-FLAG co-IP, 10 μl of anti-FLAG M2 magnetic beads (Sigma–Aldrich) were added to the cell lysate, and samples were rotated at 4°C overnight. Beads were washed with 1 ml of lysis buffer six times. For RPL7-HA and DDX21 co-IP, 2 μg of anti-HA (BioLegend), anti-DDX21 (Proteintech), mouse IgG isotype control (Invitrogen, as an HA co-IP background control), or rabbit IgG isotype control (Invitrogen, as a DDX21 co-IP background control) antibody was added to the clear cell lysate, and samples were rotated at 4°C overnight. The next day, 25 μl of Dynabeads Protein G (Invitrogen) for RPL7-HA co-IP or Protein A (Invitrogen) for DDX21 co-IP were added, and the sample was rocked for additional 4 h at 4°C. Beads were washed with 1 ml of lysis buffer six times.

For p24 co-IP in MT-2 cells, five million cells were lysed in 0.5 ml of PBS supplemented with 1% Triton and tablets of protease inhibitor (Roche). The clear lysate was incubated and rocked with 2 μg of anti-HTLV-1 p24 antibody (Santa Cruz) or mouse IgG isotype control (Invitrogen) at 4°C overnight. The following day, 25 μl of Dynabeads Protein G (Invitrogen) were added, and the sample was rotated at 4°C for additional 4 h. Beads were washed with 0.5 ml PBS three times.

After the washing step, beads were resuspended in 20 μl of lysis buffer with 5 μl of 5× protein loading dye (300 mM Tri–HCl, pH 6.8, 25% glycerol, 10% SDS, 715 mM β-ME, 0.1% bromophenol blue) and boiled for 10 min before loading onto 10% polyacrylamide denaturing gels or pre-cast NuPAGE Bis-Tris 12% polyacrylamide gels (Invitrogen). Proteins in the gels were transferred onto polyvinylidene fluoride membranes (Cytiva). Membranes were blocked with 5% non-fat milk (Bio-Rad) in 19 mM Tris–HCl, 137 mM NaCl, 27 mM KCl, and 0.1% Tween-20 (TBST) and incubated with primary antibodies at 4°C overnight. The following primary antibodies were used: anti-GFP (Invitrogen, 1:5000), anti-FLAG (Sigma–Aldrich, 1:1000), anti-HA (BioLegend, 1:2000), anti-V5 (Invitrogen, 1:1000), anti-HTLV-1 p19 (ZeptoMetrix, 1:1000), anti-HTLV-1 p24 (Santa Cruz, 1:1000), anti-RPL7 (Bethyl, 1:2000), anti-DDX21 (Proteintech, 1:1000), and anti-β-actin (Sigma–Aldrich, 1:5000). After three washes with TBST, membranes were incubated with secondary antibodies at room temperature for 1 h. The secondary antibodies used were horseradish peroxidase-conjugated goat anti-mouse (Promega, 1:5000) or goat anti-rabbit antibodies (Promega, 1:5000). The blots were developed using SuperSignal West Pico PLUS Chemiluminescent Substrate (Thermo Scientific) or Pierce Enhanced Chemiluminescence Western Blotting Substrate (Thermo Scientific) and visualized by Amersham Imager 680 imaging system (Cytiva).

## Results

### Recombinant HTLV-1 Gag was successfully purified from *E. coli*

To investigate potential proteins responsible for annealing the HTLV-1 RT primer to the highly-structured HTLV-1 PBS [17], annealing assays were initially performed using viral chaperone protein candidates HTLV-1 MA, HTLV-1 NC, and HIV-1 NC. The *in vitro*-transcribed human tRNA^Pro^ and viral genome-derived PBS domain used in this study are shown in Fig. 1A. Neither HTLV-1 MA, NC, nor the robust chaperone protein, HIV-1 NC [7], could facilitate the annealing of tRNA^Pro^ to the stable PBS domain *in vitro* (data not shown).

Because the mature NC or MA domains of HTLV-1 Gag failed to facilitate tRNA^Pro^ annealing to the PBS, we hypothesized that full-length HTLV-1 Gag may have more robust chaperone activity and facilitate tRNA^Pro^ annealing. To test this hypothesis, we successfully purified HTLV-1 Gag from *E. coli* and characterized its oligomeric state and chaperone activity *in vitro*. In our previous study, removing the C-terminal 29 highly acidic residues in HTLV-1 NC increased HTLV-1 NC’s chaperone activity, making it comparable to other retroviral NC proteins [20]. To test whether deleting these residues of HTLV-1 Gag impact its chaperone activity, HTLV-1 ∆C29 Gag was also generated and purified (Fig. 1C). The purity of the recombinant proteins was high, as shown by SDS–PAGE and Coomassie blue staining (Supplementary Fig. S1A).

Gag proteins play important roles in packaging gRNA and assembling viral particles. In HIV-1-infected cells, lower-order Gag oligomers, such as dimers or trimers, are formed in the cytoplasm, and higher-order Gag multimers assemble at the plasma membrane (PM) [32–34]. In contrast to HIV-1, there are no prominent HTLV-1 Gag–Gag interactions in the cytoplasm, and Gag oligomerization happens exclusively at the PM [33–36]. To investigate the oligomeric states of purified HTLV-1 WT and ∆C29 Gag proteins in solution, size-exclusion chromatography with multi-angle laser-light scattering analysis was performed. In the spectra shown in Supplementary Fig. S1B, only one peak was observed for each protein, corresponding to calculated molecular weights of 59.6 kD for WT and 56 kD for ∆C29. Based on the theoretical molecular weights of 48.4 and 45.3 kD for WT and ∆C29, respectively, these results indicate that HTLV-1 Gag proteins form monomers in solution, consistent with previous findings [33, 34]. To examine the RNA-binding activity of purified HTLV-1 Gag, fluorescence anisotropy (FA) assays were performed using the HTLV-1 PBS RNA and human tRNAs. *K*_d_ values in the range of 200–500 nM were obtained (Supplementary Fig. S1C–E). HTLV-1 Gag displays lower NA-binding affinity than HIV-1 Gag (*K*_d_ ∼ 73 nM, minihelix^Lys3^) [37], but higher affinity than the HTLV-1 MA and NC domains (*K*_d_ ∼ 1–3 μM, HTLV-1 gRNA-derived constructs) [17].

### HTLV-1 WT and ∆C29 Gag show weak primer annealing activity

**Figure 2.**
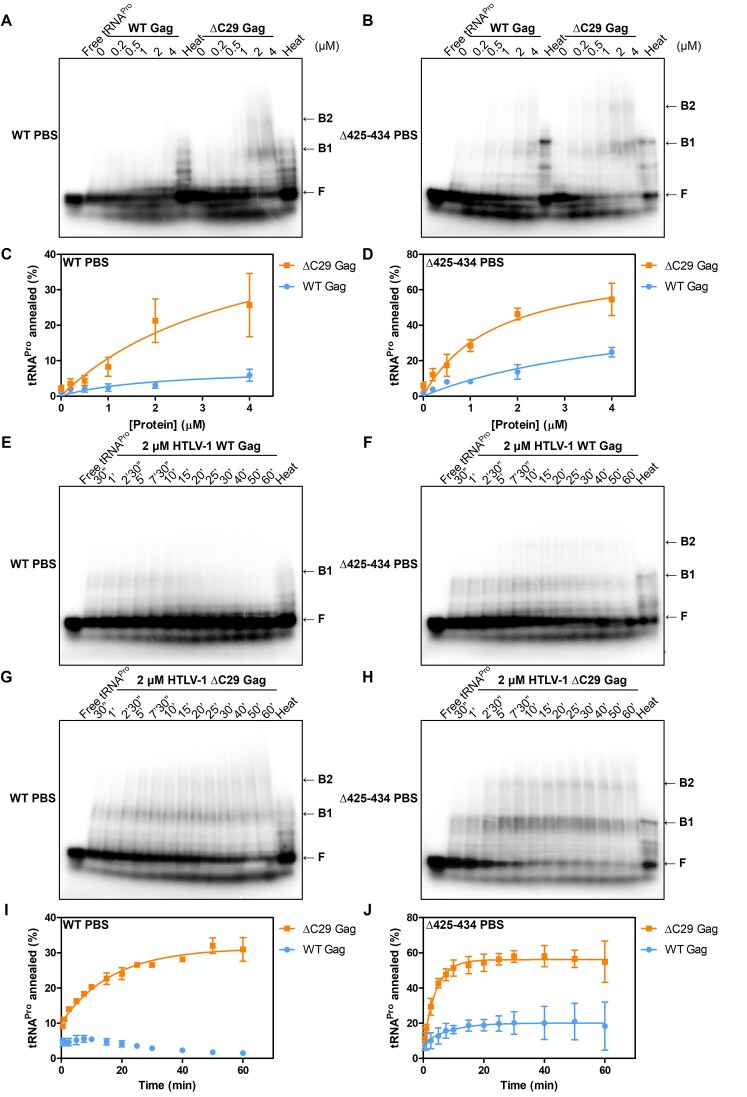
Concentration-dependence and time-course annealing assays show that HTLV-1 ∆C29 Gag chaperones tRNA^Pro^ annealing to the stable HTLV-1 WT PBS and the less structured HTLV-1 ∆425–434 PBS more effectively than HTLV-1 WT Gag. Concentration-dependence annealing assays using 20 nM 5′ ^32^P-labeled tRNA^Pro^ and 200 nM HTLV-1 WT PBS (**A**) or ∆425–434 PBS (**B**) in the presence of varying concentrations of HTLV-1 WT or ∆C29 Gag at 37°C for 1 h. F indicates free tRNA^Pro^, and B1 and B2 indicate tRNA^Pro^-PBS binary complexes with different conformations. Heat indicates the positive heat annealed control. Graphs showing percentages of tRNA^Pro^ annealed to WT (**C**) or ∆425–434 PBS (**D**) in the presence of varying concentrations of HTLV-1 WT or ∆C29 Gag. (**E**–**H**): Time-course annealing assays using 20 nM 5′ ^32^P-labeled tRNA^Pro^ and 200 nM HTLV-1 WT PBS (E and G) or ∆425–434 PBS (F and H) in the presence of 2 μM HTLV-1 WT Gag (E and F) or ∆C29 Gag (G and H) at 37°C. Graphs showing percentages of tRNA^Pro^ annealed to HTLV-1 WT PBS (**I**) or ∆425–434 PBS (**J**) at different time points in the presence of 2 μM HTLV-1 WT or ∆C29 Gag. (A, B, and E–H) Results are representative of three independent experiments. (C, D, I, and J) Lines represent exponential fits of the data with the standard deviation between three trials indicated.

To evaluate the annealing activities of HTLV-1 Gag proteins, tRNA^Pro^-PBS annealing assays were performed. Prior to annealing assays, 5′ ^32^P-labeled tRNA^Pro^ and 10-fold excess of the HTLV-1 5′ UTR PBS domain (98 nt) were folded separately and then incubated together in the presence of different concentrations of proteins at 37°C (Fig. 1A). In concentration-dependence annealing assays (Fig. 2A–D), tRNA^Pro^ was annealed to the HTLV-1 PBS in the presence of varying concentrations of HTLV-1 WT or ∆C29 Gag at 37°C for 1 h. The results are shown in Fig. 2A, where the lowest band is the free tRNA^Pro^ (F form) and the major shifted bands (B1 and B2) likely represent two different conformations of the tRNA^Pro^-PBS complex. Even in the heat-annealing positive control, very little annealed complex is formed, and the B2 band is not observed. Deletion of the C-terminal 29 acidic residues slightly improved HTLV-1 Gag’s chaperone activity, as predicted. In the presence of 4 μM HTLV-1 WT Gag, only ∼ 6% of tRNA^Pro^ is annealed to the PBS. *K*_1/2_ for WT Gag is 2 μM, and the catalytic efficiency (annealed_max_/*K*_1/2_) is 3 μM^−1^ (Fig. 2A and C; Table 1). In contrast, with 4 μM HTLV-1 ∆C29 Gag, 26% of tRNA^Pro^ is annealed to the PBS. *K*_1/2_ for ∆C29 Gag is 4 μM, and the catalytic efficiency is 6.4 μM^−1^ (Fig. 2A and C; Table 1). Based on these data, HTLV-1 ∆C29 Gag chaperoned tRNA^Pro^ annealing to the PBS ∼2-fold more efficiently than WT (Table 1).

**Table 1.** Summary of *K*_1/2_ (μM), annealed_max_ (%), catalytic efficiencies [annealed_max_/*K*_1/2_ (μM^−1^)], and fold differences of catalytic efficiencies of chaperone proteins tested in concentration-dependence annealing assays

Template RNA	Chaperone Protein	*K*_1/2_(μM)	Annealed_max_(%)	Catalytic efficiency [annealed_max_/*K*_1/2_(μM^−1^)]	Fold differences
WT PBS	WT Gag	1.99 ± 1.61	5.91 ± 1.20	2.97 ± 2.48	1.0
	∆C29 Gag	4.03 ± 2.63	25.70 ± 7.28	6.38 ± 4.54	2.2
	RPL7	0.46 ± 0.13	38.18 ± 2.01	82.99 ± 24.02	27.9
	DDX21	0.56 ± 0.17	58.26 ± 0.07	103.67 ± 31.75	34.9
∆425–434 PBS	WT Gag	4.86 ± 2.70	24.81 ± 1.90	5.11 ± 2.87	1.0
	∆C29 Gag	1.55 ± 0.42	54.55 ± 7.47	35.26 ± 10.69	6.9
	RPL7	0.39 ± 0.12	58.10 ± 6.27	150.41 ± 50.20	29.4
	DDX21	0.25 ± 0.09	68.35 ± 0.63	278.30 ± 107.40	54.5

Mean values and standard deviations were derived from three independent experiments.

To test the effect of the PBS context on annealing inhibition, we deleted nt on one side of the stem in the PBS hairpin (Fig. 1B, ∆425–434 PBS). We hypothesized that deleting this sequence would facilitate Gag-chaperoned annealing by making the PBS region less structured and more accessible. To confirm this structural modification, the secondary structure of the ∆425–434 PBS was predicted by RNAfold [38], RNAstructure [39], and mFold [40]. Structures predicted by different RNA folding programs are similar and show that the most energetically favorable conformation of the ∆425–434 PBS RNA is less structured in comparison to the WT PBS RNA (Supplementary Fig. S2). In concentration-dependence tRNA^Pro^ annealing assays with the ∆425–434 PBS and HTLV-1 WT or ∆C29 Gag, significantly more complex formation was observed compared to the WT PBS (Fig. 2A–D). This trend was also observed in the heat-annealed positive control. Band intensity for the B1 complex was more prominent for tRNA^Pro^ heat-annealed to the ∆425–434 PBS (Fig. 2B) than to the WT PBS (Fig. 2A). These results confirmed our hypothesis that the ∆425–434 PBS is more accessible, resulting in greater tRNA^Pro^ annealing. As summarized in Table 1, in the presence of 4 μM HTLV-1 WT Gag, 25% of tRNA^Pro^ is annealed to the ∆425–434 PBS, *K*_1/2_ is 5 μM, and the catalytic efficiency is 5 μM^−1^ (Fig. 2B and D; Table 1). In contrast, with 4 μM HTLV-1 ∆C29 Gag, 55% of tRNA^Pro^ is annealed to the ∆425–434 PBS, *K*_1/2_ is 1.6 μM, and the catalytic efficiency is 35 μM^−1^ (Fig. 2B and D; Table 1). Overall, HTLV-1 ∆C29 Gag chaperoned tRNA^Pro^ annealing to the ∆425–434 PBS ∼7-fold more efficiently than WT Gag (Table 1).

Time-course annealing assays were also performed in the presence of 2 μM HTLV-1 WT or ∆C29 Gag (Fig. 2E–J). After a 1-h incubation with the WT PBS, 1.5% of tRNA^Pro^ is annealed by WT Gag, whereas 31% is annealed by ∆C29 Gag (Fig. 2E, G, and I; Table 2). The scaled annealing rate (*k*′) for ∆C29 Gag, which was calculated by multiplying the annealing rate *k* by the fraction of tRNA^Pro^ annealed, is 0.019 min^−1^ (Fig. 2G and I; Table 2). The annealing rate for WT Gag could not be determined due to its low efficiency (Fig. 2E). For the ∆425–434 PBS time-course annealing assays, 18% of tRNA^Pro^ is annealed by WT Gag, whereas 55% is annealed by ∆C29 Gag (Fig. 2F, H, and J; Table 2). The scaled *k*′ values for WT and ∆C29 Gag are 0.022 min^−1^ and 0.14 min^−1^, respectively (Fig. 2F, H, and J; Table 2). The trend for time-course annealing assays is similar to that of concentration-dependence annealing assays. Overall, HTLV-1 WT or ∆C29 Gag facilitated tRNA^Pro^ annealing to the ∆425–434 PBS more efficiently than to the WT PBS. For the ∆425–434 PBS, ∆C29 Gag catalyzed tRNA^Pro^ annealing ∼6-fold faster than WT Gag (Table 2).

**Table 2.** Summary of annealing rates [*k* (min^−1^)], annealed_max_ (%), scaled annealing rates [*k′* (min^−1^)], and fold differences of scaled annealing rates of chaperone proteins tested in time-course annealing assays

Template RNA	Chaperone protein (μM)	*k*(min^−1^)	Annealed_max_(%)	*k′* (min^−1^)*	Fold differences
WT PBS	WT Gag (2)	N/A	1.52 ± 0.19	N/A	N/A
	∆C29 Gag (2)	0.060 ± 0.008	30.99 ± 2.38	0.019 ± 0.003	1.0
	RPL7 (0.8)	0.059 ± 0.008	45.05 ± 2.57	0.026 ± 0.004	1.4
	DDX21 (0.8)	0.071 ± 0.014	49.99 ± 1.51	0.036 ± 0.007	1.9
	RPL7 (0.8)+DDX21 (0.8)	0.091 ± 0.007	59.94 ± 2.31	0.055 ± 0.005	3.0
	WT Gag (2)+ RPL7 (0.8)+DDX21 (0.8)	0.160 ± 0.023	54.09 ± 0.91	0.086 ± 0.013	4.7
∆425–434 PBS	WT Gag (2)	0.120 ± 0.078	18.37 ± 9.67	0.022 ± 0.018	1.0
	∆C29 Gag (2)	0.247 ± 0.034	54.92 ± 8.33	0.136 ± 0.028	6.2
	RPL7 (0.8)	0.292 ± 0.019	60.12 ± 0.35	0.175 ± 0.011	8.0
	DDX21 (0.8)	0.487 ± 0.199	62.04 ± 6.64	0.302 ± 0.128	13.8

*The scaled annealing rate (*k*′) is calculated by multiplying *k* by annealed_max_.

Mean values and standard deviations were derived from three independent experiments.

### Affinity tagging/purification–mass spectrometry identified HTLV-1 Gag interacting partners

Although HTLV-1 ∆C29 Gag chaperoned tRNA^Pro^ annealing to the PBS more efficiently than WT Gag, this Gag truncation variant is not known to be present in HTLV-1 virions. In fact, the acidic C-terminus of HTLV-1 NC has an important functional role, preventing APOBEC3G packaging into virions and thus counteracting APOBEC3G restriction [41]. We hypothesize that host co-factors of HTLV-1 Gag may help increase its chaperone activity. To identify HTLV-1 Gag interacting partners, an AP-MS-based proteomics analysis was performed. C-terminal GFP-tagged HTLV-1 Gag and an empty GFP expression vector (as a separate pull-down background control) were expressed in HEK293T cells. This vector encodes a Twin-Strep tag at the C-terminus of GFP, which is pulled down by streptactin (Fig. 1D). Using this strategy, both GFP and HTLV-1 Gag-GFP were significantly enriched in the pull-down fractions (Supplementary Fig. S3), and co-factors were identified by MS.

A list of all protein hits can be found in Supplementary Table S3, and the top 10 hits are listed in Table 3. Among the top hits, six are ribosomal proteins (RPL4, RPL7A, RPL6, RPL7, RPS3A, and RPL18), two are protein glycosyltransferases (RPN1 and DDOST), two are NA chaperone proteins/helicases (RPL7 and DDX21), and one is fatty acid peroxisomal importer protein (ABCD3). Both RPN1 and DDOST are subunits in the oligosaccharyltransferase (OST) complex, which is located in the rough ER and essential for N-linked glycosylation on asparagine residues [42]. RPL7 has been reported to be packaged into HIV-1 virions and to interact with the NC domain of HIV-1 Gag to synergistically increase annealing of ${\mathrm{tRNA}}_{{\mathrm{UUU}}}^{{\mathrm{Lys}}3}$ to the PBS [21, 22]. DDX21 is an ATP-dependent helicase and displays helix-unwinding and RNA-folding activities [43, 44]; it was shown to interact with HIV-1 Rev protein through the DEAD domain and thereby increase Rev-Rev response element (RRE) binding affinity [28]. Because our goal was to identify potential RNA chaperone proteins and/or helicases that may help increase HTLV-1 Gag’s chaperone activity, we picked RPL7 and DDX21 for validation and further investigation as potential tRNA-annealing factors.

**Table 3.** List of top 10 AP-MS protein hits

Gene symbol	Protein name	Total spectrum count	
		Gag-GFP	GFP
RPN1	Dolichyl-diphosphooligosaccharide— protein glycosyltransferase subunit 1	50	4
RPL4	60S ribosomal protein L4	48	1
ABCD3	ATP-binding cassette sub-family D member 3	37	4
RPL7A	60S ribosomal protein L7a	34	2
RPL6	60S ribosomal protein L6	31	0
**RPL7**	**60S ribosomal protein L7**	**25**	**0**
RPS3A	40S ribosomal protein S3a	23	3
**DDX21**	**Nucleolar RNA helicase 2**	**22**	**0**
RPL18	60S ribosomal protein L18	20	0
DDOST	Dolichyl-diphosphooligosaccharide— protein glycosyltransferase 48 kDa subunit	20	1

### Interactions of HTLV-1 Gag with RPL7 and DDX21 are validated and independent of RNA binding

**Figure 3.**
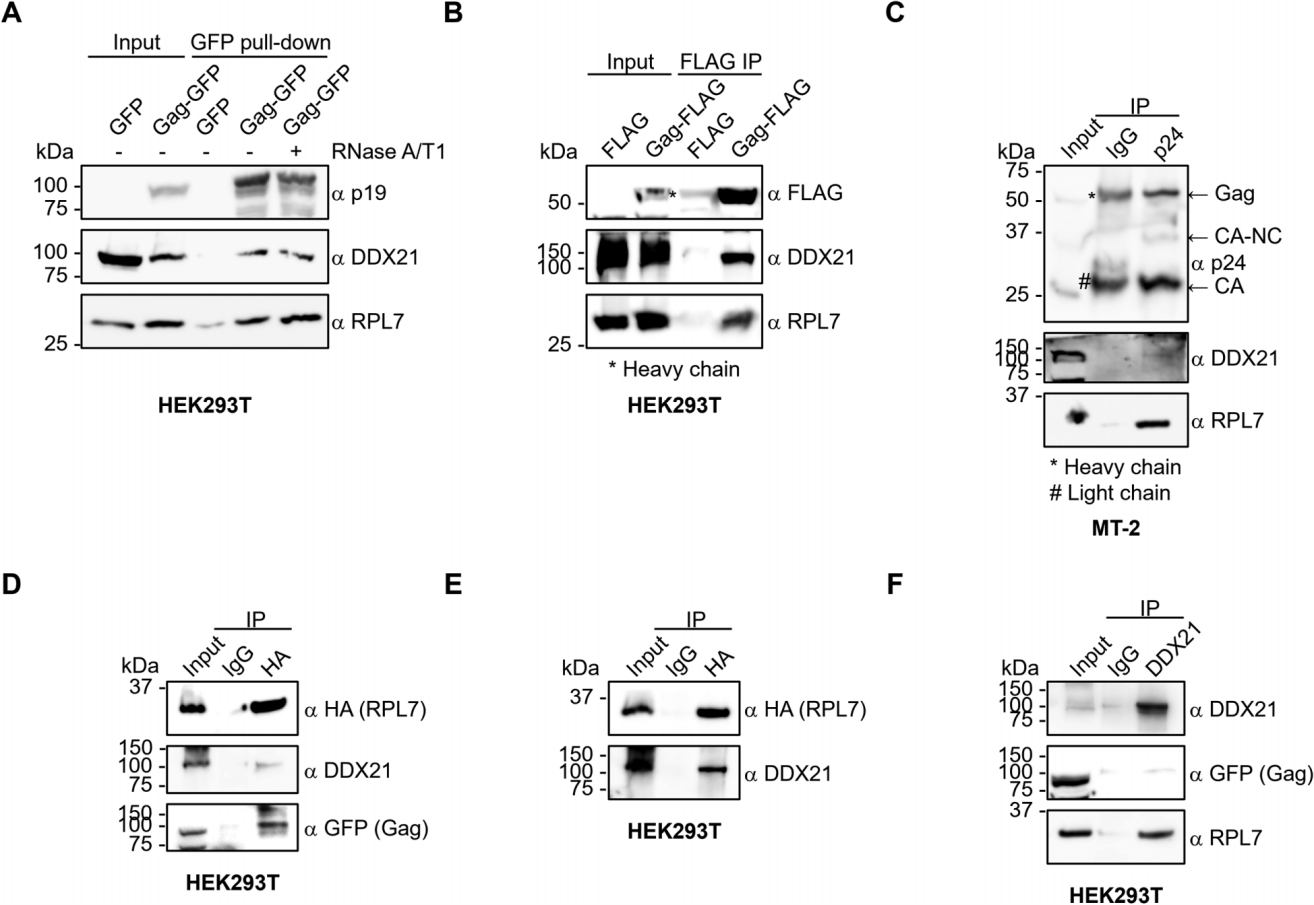
HTLV-1 Gag interacts with RPL7 and DDX21 in an RNA-independent manner as analyzed by pull-down and reciprocal co-IP. (**A**) Western blot analysis of HEK293T cell lysates overexpressing GFP or HTLV-1 Gag-GFP with or without RNase treatment. Proteins were pulled down by streptactin and immunoblotted using anti-HTLV-1 p19 (MA), anti-DDX21, and anti-RPL7 antibodies. (**B**) FLAG or HTLV-1 Gag-FLAG expressed in HEK293T cell lysate was IP’d using an anti-FLAG antibody. Co-IP’d proteins were immunoblotted using anti-DDX21 and anti-RPL7 antibodies. (**C**) Gag, CA-NC, and CA in MT-2 cell lysate were IP’d using an anti-HTLV-1 p24 (CA) antibody. Co-IP’d proteins were immunoblotted using anti-DDX21 and anti-RPL7 antibodies. RPL7-HA was IP’d using an anti-HA antibody in lysate from HEK293T cells with (**D**) or without (**E**) HTLV-1 Gag-GFP co-overexpression. Co-IP'd proteins were immunoblotted using anti-DDX21 and anti-GFP antibodies. (**F**) DDX21 was IP’d by an anti-DDX21 antibody in lysate from HEK293T cells overexpressing HTLV-1 Gag-GFP. Co-IP’d proteins were immunoblotted using anti-GFP and anti-RPL7 antibodies. Results are representative of at least three independent experiments.

To confirm the AP-MS results, HTLV-1 Gag-GFP pull-down and HTLV-1 Gag-FLAG co-IP followed by immunoblotting were performed in HEK293T cell lysate. When HTLV-1 Gag-GFP, but not GFP, was pulled-down, RPL7 and DDX21 were co-pulled-down (Fig. 3A). When HTLV-1 Gag-FLAG, but not FLAG, was IP’d, RPL7 and DDX21 were co-IP’d (Fig. 3B). Both RPL7 and DDX21 are RNA-binding proteins [28, 45]. To investigate whether the interactions of HTLV-1 Gag with RPL7 and DDX21 are direct or mediated by RNA, cell lysate was treated with RNase A/T1 before Gag-GFP pull-down assays. We found that the levels of RPL7 and DDX21 co-pulled-down with Gag-GFP were not impacted by RNase treatment, suggesting the interactions are RNA-independent (Fig. 3A). To further confirm the interaction, a reciprocal co-IP was performed in cell lysate from HEK293T cells co-overexpressing Gag-GFP and HA-RPL7 (Fig. 1D and F); when HA-RPL7 was IP’d, Gag-GFP and DDX21 were co-IP’d. No interaction was observed in the IgG isotype background control sample (Fig. 3D). To rule out the possibility that RPL7 interacts with DDX21 through Gag, HA-RPL7 co-IP was performed in HEK293T cell lysate without Gag expression. DDX21 was co-IP’d with HA-RPL7 in the absence of Gag (Fig. 3E). A reciprocal co-IP in HEK293T cells overexpressing Gag-GFP was performed; DDX21 pulled down by an anti-DDX21 antibody co-IP’d with Gag-GFP and RPL7 (Fig. 3F). The above results were obtained from exogenously overexpressed Gag in HEK293T cells. To confirm the interactions at endogenous expression levels, co-IPs were carried out in a more physiologically relevant chronically HTLV-1-infected MT-2 cell line. When Gag, CA-NC, and CA were pulled down using an anti-p24 antibody, RPL7 and DDX21 were co-IP’d (Fig. 3C). Overall, these results confirm the interactions of HTLV-1 Gag with DDX21 and RPL7 and are consistent with the AP-MS data. In addition, RPL7 and DDX21 also interact with each other, indicating that Gag, RPL7, and DDX21 may form a complex.

HTLV-1 Gag is essential for assembling viral particles and packaging gRNA into virions. In addition to specifically binding to gRNA, two other features of Gag, myristoylation and oligomerization, are required for efficient gRNA packaging and viral assembly. Co-translational myristoylation of Gly2 in the MA domain of Gag is required for Gag anchoring to the PM [34, 46, 47]. Gag oligomerization occurs on or near the PM through N-terminal CA domain interactions [48]. Major residues involved in oligomerization are Met147 and Tyr191, located in the CA dimer interface, and Gln177 and Phe178, located in the CA trimer interface [49]. To test the role of myristoylation, Gly2 was changed to Ala (G2A). To disrupt Gag oligomer formation, M147AY191A and Q177AF178A mutants were made. We observed that the levels of RPL7 and DDX21 co-IP’d with Gag-FLAG were not changed significantly in these myristoylation- and oligomerization-deficient Gag mutants in comparison to WT Gag (Supplementary Fig. S4). Taken together, these results demonstrate that interactions of HTLV-1 Gag with RPL7 and DDX21 are direct and independent of the presence of RNA, Gag membrane binding, and Gag multimerization.

### The HTLV-1 Gag NC zinc finger structures are essential for the interactions with RPL7 and DDX21

**Figure 4.**
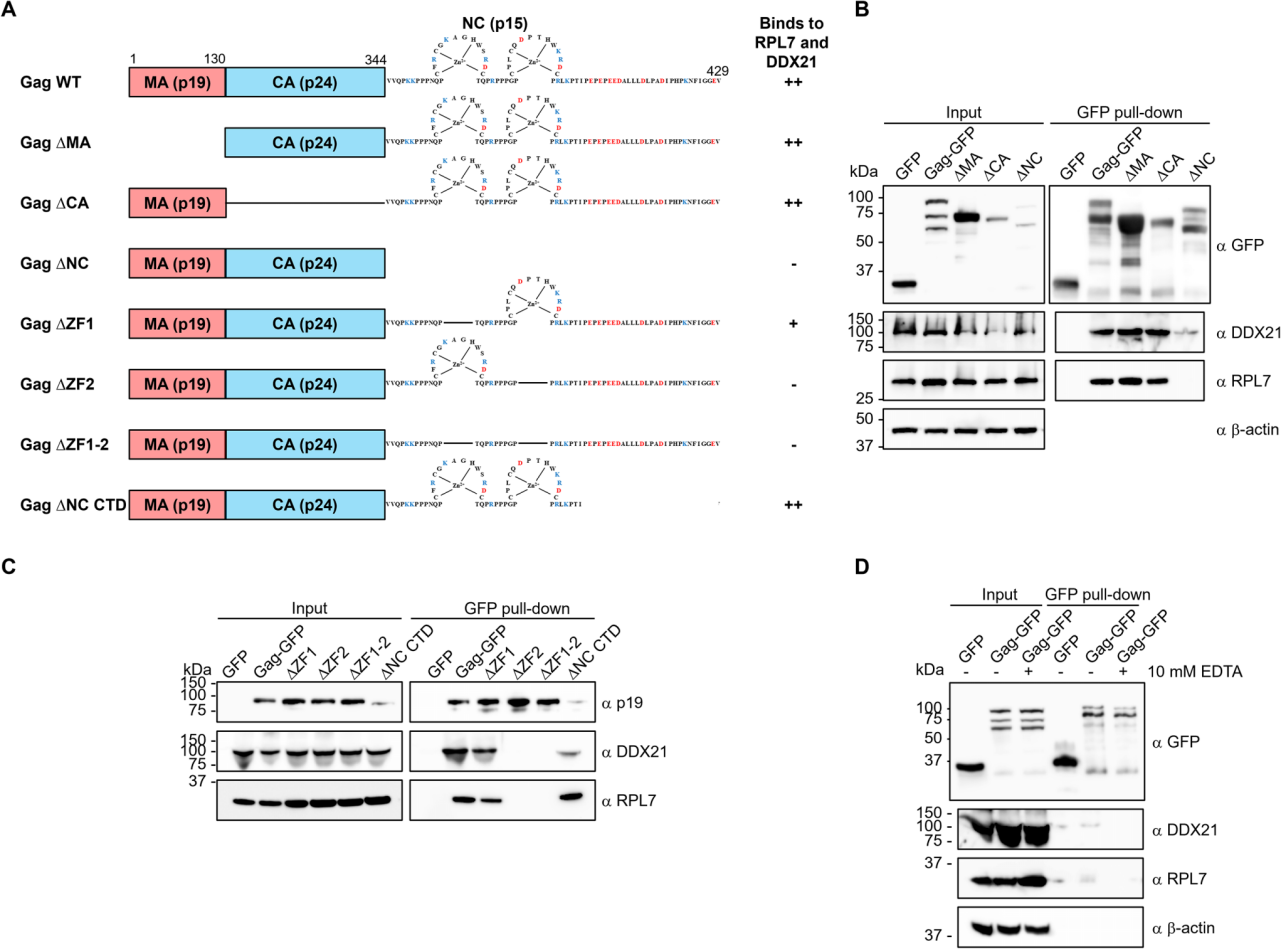
ZF structures in the NC domain of HTLV-1 Gag are essential for the interactions with DDX21 and RPL7. (**A**) Schematic representation of HTLV-1 Gag domain and NC subdomain truncation constructs used in the pull-down study. (++), (+), and (−) indicate the relative ability of each construct to co-pull-down DDX21 and RPL7. (**B**) GFP, WT Gag-GFP, ∆MA, ∆CA, and ∆NC Gag-GFP in HEK293T cell lysate were pulled down by streptactin and immunoblotted using an anti-GFP antibody. Co-pulled-down proteins were immunoblotted using anti-DDX21 and anti-RPL7 antibodies. (**C**) GFP, WT Gag-GFP, ∆ZF1, ∆ZF2, ∆ZF1-2, and ∆NC CTD Gag-GFP in HEK293T cell lysate were pulled down by streptactin and immunoblotted using an anti-HTLV-1 p19 antibody. Co-pulled-down proteins were immunoblotted using anti-DDX21 and anti-RPL7 antibodies. (**D**) Cell lysate from HEK293T cells overexpressing HTLV-1 Gag-GFP was treated with or without 10 mM EDTA. GFP or HTLV-1 Gag-GFP was pulled down by streptactin and immunoblotted using an anti-GFP antibody. Co-pulled-down proteins were immunoblotted using anti-DDX21 and anti-RPL7 antibodies. β-actin was used as a loading control. Results are representative of three independent experiments.

We next investigated which domains and subdomains of HTLV-1 Gag interact with RPL7 and DDX21. Single-domain truncation mutants of Gag-GFP (∆MA, ∆CA, and ∆NC) were prepared and transfected into HEK293T cells, followed by Gag-GFP pull-down and immunoblotting (Fig. 4A). The interactions of Gag with RPL7 and DDX21 were significantly diminished only upon deletion of the NC domain (Fig. 4B). To map the NC subdomain(s) that interact with RPL7 and DDX21, single- or double-subdomain zinc finger (ZF) mutants, ∆ZF1, ∆ZF2, ∆ZF1-2, and an NC CTD deletion (∆NC CTD) were made and transfected into HEK293T cells, followed by pull-down assays (Fig. 4A). In comparison to the interaction of WT Gag with RPL7 and DDX21, the interaction was slightly reduced when ZF1 in Gag was deleted and was more severely impaired when ZF2 or both ZF1 and ZF2 were absent (Fig. 4C). These results indicate that ZF2 is more important than ZF1 for the interaction and that HTLV-1 Gag interacts with RPL7 and DDX21 through both ZFs. Deleting NC’s CTD did not significantly affect the interaction.

The coordination of Zn^2+^ is crucial for maintaining NC’s structure [50]. To investigate the importance of ZF structures on the interaction, 10 mM EDTA was added to the cell lysate as a Zn^2+^ chelator prior to performing Gag-GFP pull-down assays (Fig. 4D). The interactions of Gag-GFP with RPL7 and DDX21 were abolished upon EDTA treatment suggesting that the ZF structures in the NC domain of HTLV-1 Gag are indispensable for interacting with RPL7 and DDX21.

### The N-terminal basic leucine zipper domain and CTD of RPL7 interact with HTLV-1 Gag and DDX21

**Figure 5.**
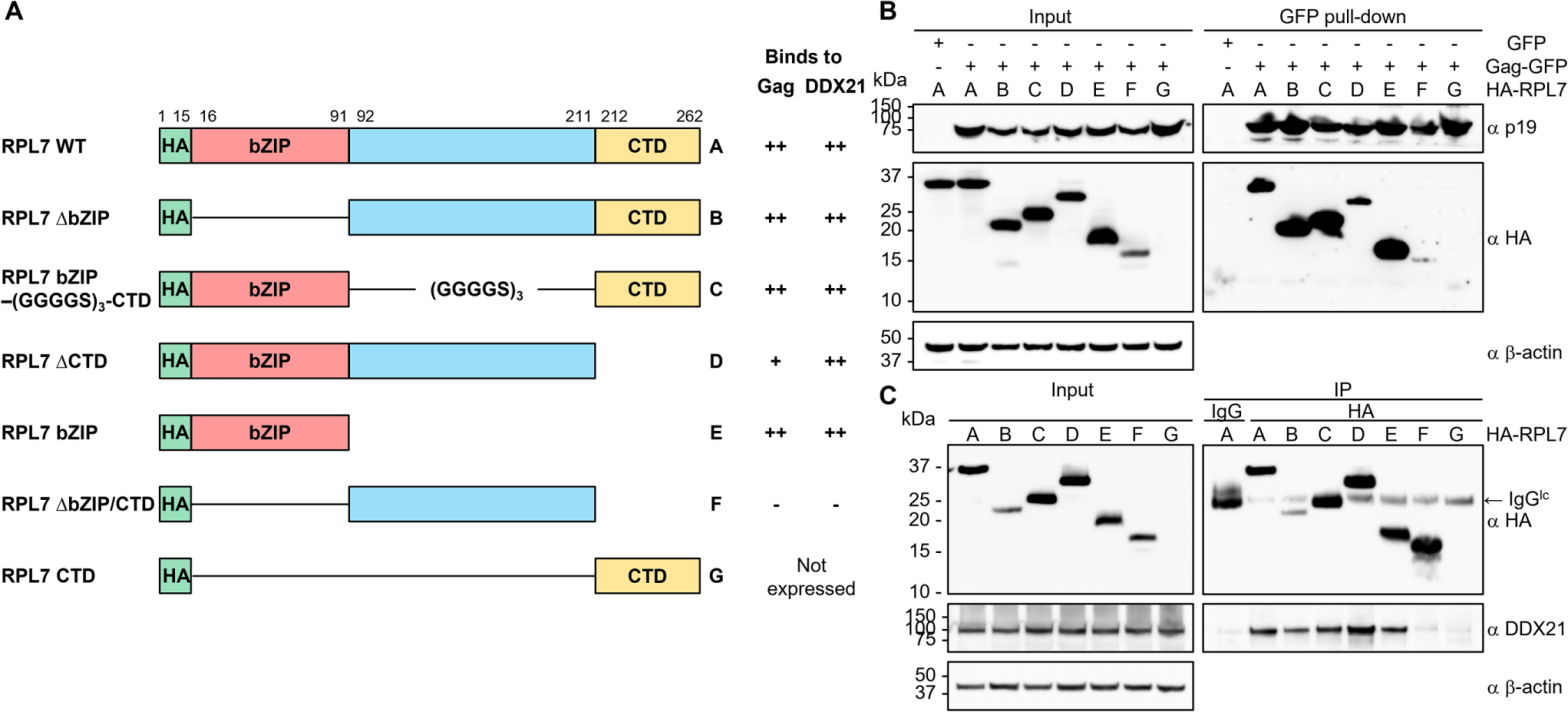
RPL7 interacts with HTLV-1 Gag and DDX21 through the N-terminal basic leucine zipper (b-ZIP) domain and CTD. (**A**) Schematic representation of human RPL7 WT and domain truncation constructs used in this study. Letters on the right side indicate the construct used in each lane in panels (B) and (C). Relative binding of RPL7 variants to HTLV-1 Gag and DDX21 are shown as (++), (+), or (−). (**B**) GFP or Gag-GFP was pulled down by streptactin and immunoblotted using an anti-HTLV-1 p19 antibody in cell lysate from HEK293T cells co-transfected with HTLV-1 Gag-GFP and WT or truncation mutant constructs of HA-RPL7. Co-pulled-down HA-RPL7 was immunoblotted using an anti-HA antibody. (**C**) RPL7-HA WT or truncation mutants were IP’d and immunoblotted using anti-HA antibody. Co-IP’d DDX21 was immunoblotted using anti-DDX21 antibody. (B, C) β-actin was used as a loading control. Results are representative of three independent experiments.

We next probed which domains in RPL7 are involved in HTLV-1 Gag and DDX21 interactions. RPL7 is composed of the N-terminal basic leucine-zipper domain (bZIP, residues 1–77), the C-terminal NA-binding domain (CTD, residues 198–248), and the central globular domain (residues 78–197) (Fig. 5A) [45, 51]. The CTD lacks a canonical NA-binding domain, and bZIP and CTD have different preferences for RNA binding [45, 51, 52]. The bZIP domain has a higher binding affinity to messenger RNA (mRNA) than 28S ribosomal RNA (rRNA) [51], whereas the opposite is true for the CTD [45]. Other known RPL7 cellular interacting partners include ribosomal protein S7, zinc finger protein 7, and vitamin D receptor. RPL7 interacts with all of them through the bZIP domain [53, 54]. To map which domains in RPL7 interact with HTLV-1 Gag or DDX21, a series of single-domain (Fig. 5A, constructs B to D) or double-domain (Fig. 5A, constructs E to G) truncation mutants were prepared. In mutant C, the (GGGGS)_3_ flexible linker was added between the N-terminal bZIP and CTD to separate these two domains in order to minimize interfering with domain folding (Fig. 5A, construct C) [55]. Mutant G, which contains the coding sequence for the C-terminal 51 residues, failed to express in HEK293T cells; thus, we were unable to assess its influence on the interactions (Fig. 5B and C).

Gag-GFP and WT or truncation mutant constructs of HA-RPL7 were co-transfected into HEK293T cells and Gag-GFP pull-down assays were performed (Fig. 5B). In comparison to the amount of WT HA-RPL7 co-pulled-down with Gag-GFP, the interaction was slightly decreased in mutant D lacking the CTD and severely impaired in mutant F when both NA-binding domains were deleted (Fig. 5B). These results show that CTD plays a more crucial role than bZIP for the interaction. However, bZIP also participates in the interaction, as a construct lacking both bZIP and CTD resulted in a more severely impaired interaction compared with the single-domain CTD deletion. To examine which domains in RPL7 interact with DDX21, WT or truncation mutant constructs of HA-RPL7 were transfected into HEK293T cells, and HA-RPL7 co-IP was carried out. Similar to results obtained with the HTLV-1 Gag/HA-RPL7 study, the interaction between DDX21 and RPL7 was severely impaired with the ∆bZIP/CTD RPL7 mutant (Fig. 5C, construct F). Taken together, both NA-binding domains, bZIP and CTD, in RPL7 interact with HTLV-1 Gag and DDX21.

### Helicase core and C-terminal domains of DDX21 interact with HTLV-1 Gag

**Figure 6.**
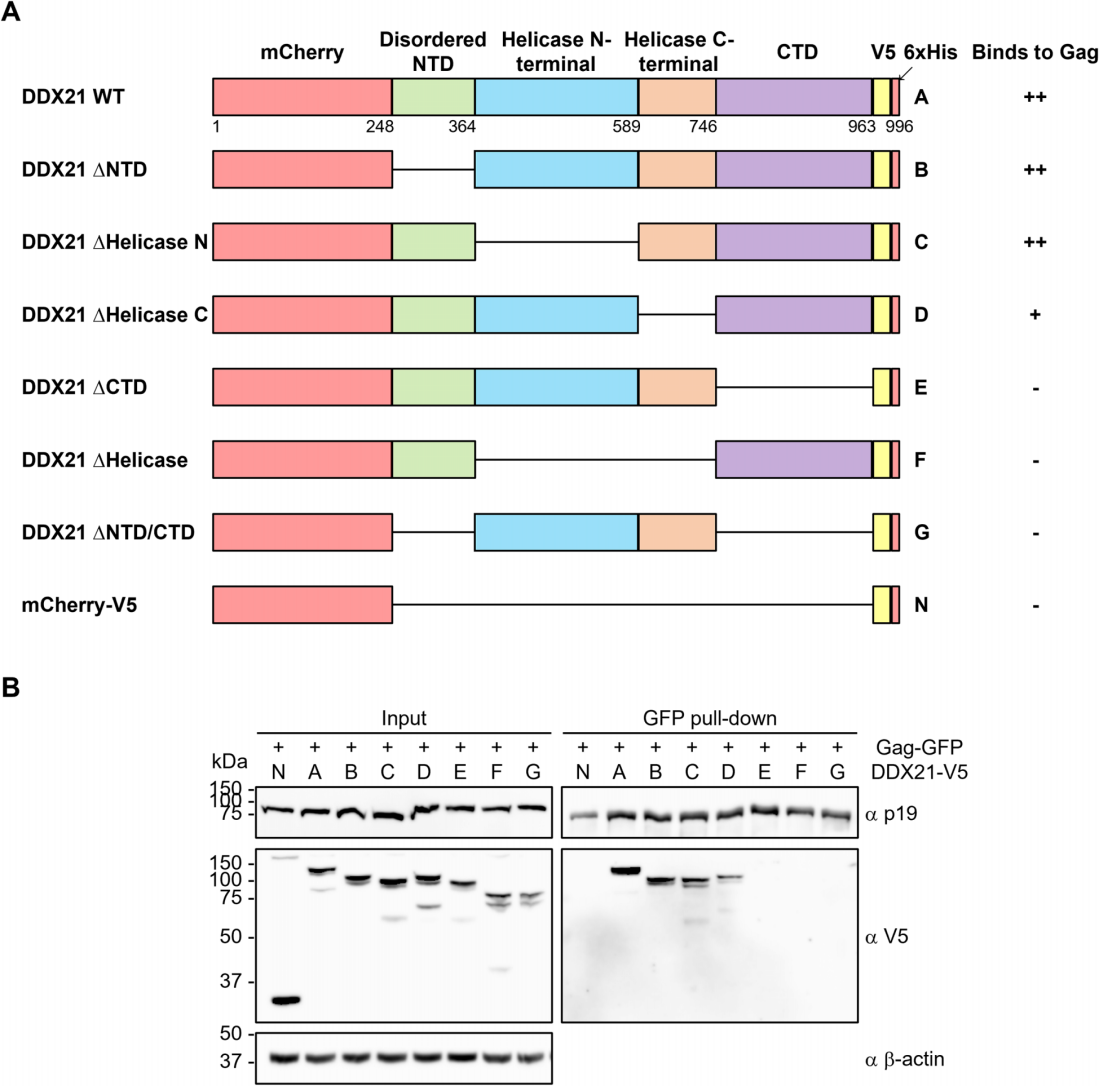
DDX21 interacts with HTLV-1 Gag through the helicase core and the CTD. (**A**) Schematic representation of human DDX21 WT and domain truncation constructs used in the pull-down study. Letters on the right represent the construct used in each lane in panel (B). Relative binding of DDX21 variants to HTLV-1 Gag are reported as (++), (+), or (−). (**B**) Gag-GFP was pulled down by streptactin and immunoblotted using an anti-HTLV-1 p19 antibody in cell lysate from HEK293T cells co-transfected with HTLV-1 Gag-GFP and WT or truncation mutant constructs of mCherry-DDX21-V5. Co-pulled-down DDX21-V5 was immunoblotted using an anti-V5 antibody. β-actin was used as a loading control. Results are representative of three independent experiments.

DDX21 is a member of the DEAD-box helicase family. The domain organization of DDX21 includes a disordered NTD (residues 1–116), an ATP-binding DEAD-box helicase NTD (helicase N, residues 117–341), a helicase CTD (helicase C, residues 342–498), and a disordered C-terminal foldase domain (CTD, residues 499–715) (Fig. 6A) [43]. DDX21 displays two distinct enzymatic activities; its ATP-dependent RNA-unwinding activity requires the disordered N-terminus and the core helicase domain, while its RNA folding activity requires the C-terminal foldase domain [43, 44]. To map which domains in DDX21 interact with HTLV-1 Gag, single-domain (Fig. 6A, constructs B to E) and double-domain (Fig. 6A, constructs F and G) deletion mutants were prepared. HTLV-1 Gag-GFP and WT or deletion mutants of mCherry-DDX21-V5 were co-transfected into HEK293T cells and analyzed in Gag-GFP pull-down studies. In comparison to the amount of WT DDX21 co-pulled-down, the interaction was slightly decreased in mutant D lacking the helicase CTD and completely abolished in mutant F wherein the entire helicase core domain was missing (Fig. 6B). These results indicate that the presence of the helicase core is essential for the interaction, and that the helicase CTD is more important for interacting with Gag than the helicase NTD. In addition, the interaction disappeared when the DDX21 CTD was absent (Fig. 6B, construct E). Collectively, DDX21 interacts with HTLV-1 Gag through the helicase core domain and the CTD.

### RPL7 and DDX21 are packaged into HTLV-1 virions

**Figure 7.**
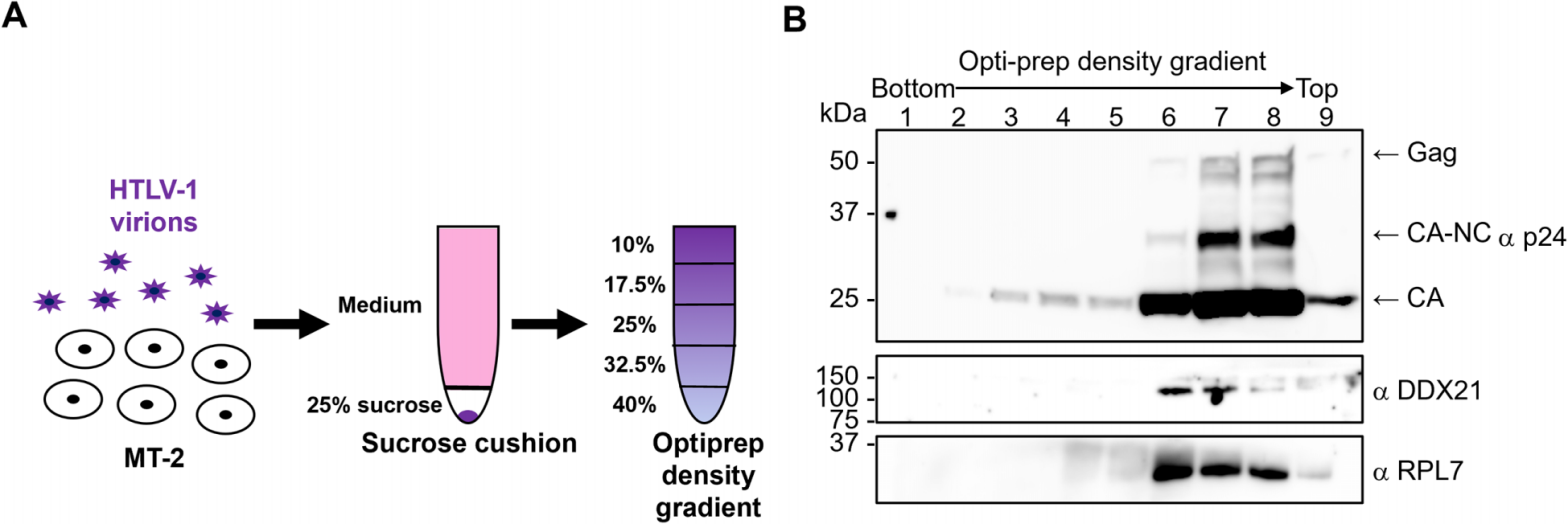
RPL7 and DDX21 are packaged into HTLV-1 virions. (**A**) Schematic diagram of purification of HTLV-1 virus from MT-2 cell culture medium through a sucrose cushion and further fractionation of the viral pellet through an OptiPrep density gradient. (**B**) Fractionated viral lysate was immunoblotted using anti-HTLV-1 p24, anti-DDX21, and anti-RPL7 antibodies. Results are representative of three independent experiments.

To investigate whether these Gag-interacting partners are packaged into HTLV-1 particles, virions purified by sucrose cushion were fractionated using an OptiPrep density gradient to remove secreted proteins, exosomes, and cytoplasmic contaminants (Fig. 7A) [56]. As shown in Fig. 7B (top), HTLV-1 proteins were present in fractions 6–9. Both RPL7 and DDX21 co-sedimented with CA and Gag in these same fractions (Fig. 7B, middle and bottom).

### Human DDX21 and RPL7 facilitate tRNA^Pro^ annealing to the HTLV-1 PBS more efficiently than HTLV-1 WT and ∆C29 Gag

We next purified RPL7 and DDX21 proteins and characterized their ability to promote tRNA^Pro^ annealing to the HTLV-1 PBS. WT and mutant RPL7 lacking one or both NA-binding domains and WT DDX21 were purified from *E. coli* (Supplementary Fig. S5A and B). To confirm that purified WT RPL7 protein was folded properly (since denaturing conditions during purification were required), circular dichroism (CD) was performed. The spectrum shows two minima at 208 and 222 nm, corresponding to the characteristic signals for α-helical proteins (Supplementary Fig. S5C) [57]. Analysis of the CD spectrum by the CAPITO algorithm indicated ∼37% α-helical and ∼5% β-strand character [58]. This result is consistent with the α-helix-rich RPL7 structure and scattered β-strand structures in the middle globular domain [59]. To examine the RNA binding activity of purified RPL7 and DDX21, FA assays were performed using the HTLV-1 PBS RNA and human tRNAs. *K*_d_ values in the range of 150–300 nM for RPL7 and 20–75 nM for DDX21 were obtained (Supplementary Fig. S5D–I). For RPL7, the observed values are consistent with a previous study using saturation filter binding assays showing that RPL7 binds to rRNA and mRNA (*K*_d_ ∼ 20 nM) with higher affinity than to tRNA [45, 51]. For DDX21, the values we obtained are similar to those previously reported for single-stranded RNA (*K*_d_ ∼ 31 nM), double-stranded RNA (dsRNA, *K*_d_ ∼ 10 nM) [60], and HIV-1 RRE RNA (*K*_d_ ∼ 37 nM) [28]. DDX21 exhibits significantly higher affinity to the HTLV-1 PBS RNA and human tRNAs in comparison to HTLV-1 Gag and RPL7. In addition to the RNA binding activity, the ATPase activity of DDX21 was examined using malachite green phosphate assays. The ATP hydrolysis rate of DDX21 is 5.93 ± 0.38 min^−1^ protein^−1^ (Supplementary Fig. S5J). This observed value is within the published range for DEAD-box helicases [28, 61].

**Figure 8.**
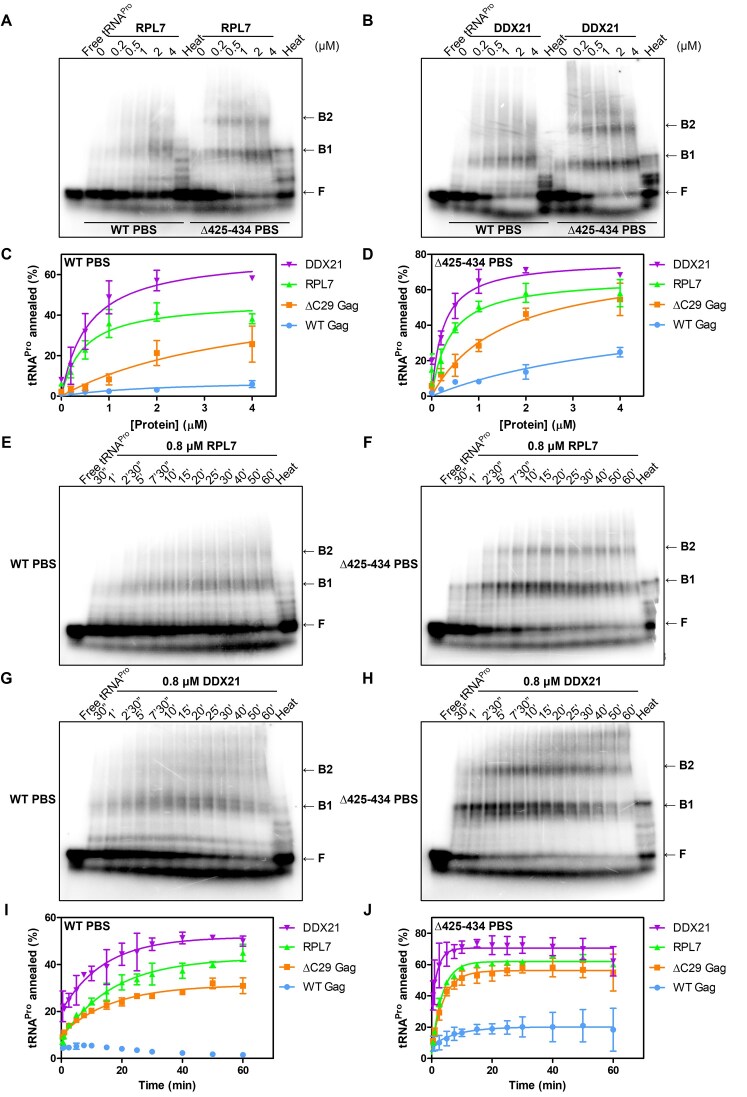
Concentration-dependence and time-course annealing assays show that human DDX21 and RPL7 facilitate tRNA^Pro^ annealing to the HTLV-1 PBS more efficiently than HTLV-1 WT and ∆C29 Gag. Concentration-dependence annealing assays using 20 nM 5′ ^32^P-labeled tRNA^Pro^ and 200 nM HTLV-1 WT PBS or ∆425–434 PBS in the presence of varying concentrations of RPL7 (**A**) or DDX21 (**B**) at 37°C for 1 h. F indicates free tRNA^Pro^, and B1 and B2 indicate different tRNA^Pro^-PBS binary complexes. Heat indicates heat annealing, the positive control. Graphs showing percentages of tRNA^Pro^ annealed to WT (**C**) or ∆425–434 PBS (**D**) in the presence of varying concentrations of HTLV-1 WT Gag, ∆C29 Gag, RPL7, or DDX21. (**E**–**H**) Time-course annealing assays using 20 nM 5′ ^32^P-labeled tRNA^Pro^ and 200 nM HTLV-1 WT PBS (E and G) or ∆425–434 PBS (F and H) in the presence of 0.8 μM RPL7 (E and F) or DDX21 (G and H) at 37°C. Graphs showing percentages of tRNA^Pro^ annealed to HTLV-1 WT PBS (**I**) or ∆425–434 PBS (**J**) at different time points in the presence of 2 μM HTLV-1 WT Gag, 2 μM ∆C29 Gag, 0.8 μM RPL7, or 0.8 μM DDX21. (A, B, and E–H) Results are representative of three independent experiments. (C, D, I, and J) Lines represent exponential fits of the data with the standard deviation between three trials indicated.

To characterize the chaperone activities of RPL7 and DDX21, tRNA^Pro^ annealing assays were performed with the same RNA constructs shown in Fig. 1A and B. In concentration-dependence annealing assays carried out for a period of 60 min (Fig. 8A–D), RPL7 and DDX21 facilitate tRNA^Pro^ annealing to the PBS more efficiently than HTLV-1 WT or ∆C29 Gag (compare Fig. 2A and B with Fig. 8A–D). With 4 μM chaperone protein and the WT PBS, RPL7 promoted 38% annealed complex while DDX21 achieved 58% complex formation (Fig. 8A–C). *K*_1/2_ for RPL7 is 0.46 μM and the catalytic efficiency (annealed_max_/*K*_1/2_) is 83 μM^−1^ (Table 1). For DDX21, *K*_1/2_ is 0.56 μM and the catalytic efficiency is 104 μM^−1^ (Table 1). With 4 μM chaperone protein and the less structured ∆425–434 PBS, RPL7 promoted 58% tRNA^Pro^ annealed complex whereas 68% of tRNA^Pro^ is annealed by DDX21 (Fig. 8A, B, and D). *K*_1/2_ for RPL7 is 0.39 μM and the catalytic efficiency is 150 μM^−1^ (Table 1). For DDX21, *K*_1/2_ is 0.25 μM and the catalytic efficiency is 278 μM^−1^ (Table 1). With these proteins, especially DDX21 with the mutant PBS domain, bands that migrate above the major B2 complex are observed, suggesting they can promote additional conformational states. In summary, DDX21 chaperoned tRNA^Pro^ annealing 1.3- and 16-fold more efficiently to the WT PBS and 1.9- and 7.9-fold more efficiently to the ∆425–434 PBS compared to RPL7 and ∆C29 Gag, respectively. RPL7 facilitated tRNA^Pro^ annealing 13-fold more efficiently to the WT PBS and 4.3-fold more efficiently to the ∆425–434 PBS compared to ∆C29 Gag (Table 1).

Time-course annealing assays were next performed in the presence of 0.8 μM RPL7 or DDX21; reactions were terminated at different time points up to 1 h (Fig. 8E–H). After a 1-h incubation with the WT PBS, 45% of tRNA^Pro^ was annealed by RPL7, while 50% of tRNA^Pro^ was annealed by DDX21. The scaled annealing rates (*k′*) are 0.026 and 0.036 min^−1^, respectively (Fig. 8E, G, and I; Table 2). For the ∆425–434 PBS time-course annealing assays, 60% of tRNA^Pro^ was annealed by RPL7, while 62% of tRNA^Pro^ was annealed by DDX21. The *k*′ values for RPL7 and DDX21 are 0.18 and 0.30 min^−1^ (Fig. 8F, H, and J; Table 2). The trend for time-course annealing assays was similar to that of concentration-dependence assays; 0.8 μM RPL7 and DDX21 chaperoned tRNA^Pro^ annealing to the ∆425–434 PBS more efficiently than to the WT PBS. Overall, DDX21 has the strongest chaperone activity in comparison to RPL7 and ∆C29 Gag, and RPL7 shows better chaperone activity than ∆C29 Gag. DDX21 facilitated tRNA^Pro^ annealing 1.4- and 1.9-fold faster to the WT PBS and 1.7- and 2.2-fold faster to the ∆425–434 PBS compared to RPL7 and ∆C29 Gag, respectively. RPL7 chaperoned tRNA^Pro^ annealing 1.4-fold faster to the WT PBS and 1.3-fold faster to the ∆425–434 PBS compared to ∆C29 Gag (Table 2).

### Both bZIP and CTD of RPL7 are involved in chaperoning the annealing of tRNA^Pro^ to the HTLV-1 PBS

To determine which domains in RPL7 play a role in tRNA^Pro^ annealing to the PBS, RPL7 variants lacking one or both of the NA-binding domains, ∆bZIP, ∆CTD, or ∆bZIP/CTD, were purified and tested in concentration-dependence annealing assays (Supplementary Figs S5A and S6). In the presence of 1 μM RPL7 proteins and the less structured ∆425–434 PBS, 46% tRNA^Pro^ is annealed by WT, 23% by ∆bZIP, 44% by ∆CTD, and 12% by the ∆bZIP/CTD variant (Supplementary Fig. S6A and B). Thus, WT and ∆CTD RPL7 have similar chaperone activity, whereas the annealing activity of the ∆bZIP protein is slightly impaired; the activity is further reduced when both bZIP and CTD are deleted, showing a non-additive positive epistatic effect on enzyme activity.

### RPL7 and DDX21 synergistically chaperone the annealing of tRNA^Pro^ to the HTLV-1 PBS

To investigate possible synergistic effects of the two Gag-binding proteins, time-course annealing assays were performed (Supplementary Figs S7–S9). In the presence of only one protein, after 1 h, 50% of tRNA^Pro^ is annealed to the WT PBS domain by 0.8 μM DDX21 (Fig. 8G and I), 45% by 0.8 μM RPL7 (Fig. 8E and I), and 1.5% by 2 μM WT Gag (Fig. 2E and I). When 0.8 μM RPL7 and 2 μM HTLV-1 WT Gag are in the same reaction, 37% of tRNA^Pro^ is annealed (Supplementary Fig. S7A and B). Although annealing activity was greater in reactions containing both RPL7 and Gag than with WT Gag alone, it was similar to RPL7 alone, indicating the absence of a synergistic effect on tRNA^Pro^ annealing to the WT PBS.

When DDX21 and HTLV-1 WT Gag are both present at 0.8 μM, 38% of tRNA^Pro^ is annealed. This level drops to 29% when the Gag concentration is increased to 2 μM (Supplementary Fig. S8A–C). This indicates the absence of a synergistic effect on the annealing of tRNA^Pro^ to the WT PBS and possible competitive binding of these proteins to the substrate.

When RPL7 and DDX21 are in the same reaction at 0.8 μM, 60% of tRNA^Pro^ is annealed and the scaled annealing rate (*k*′) is 0.055 min^−1^ (Supplementary Fig. S9A and B; Table 2). This rate is 2.1- and 1.5-fold greater than RPL7 or DDX21 alone, respectively, indicating a synergistic effect of tRNA^Pro^ annealing to the WT PBS (Table 2).

### When RPL7, DDX21, and HTLV-1 Gag are all present, the annealing of tRNA^Pro^ to the HTLV-1 PBS is more efficient than with a single protein or combinations of two proteins

**Figure 9.**
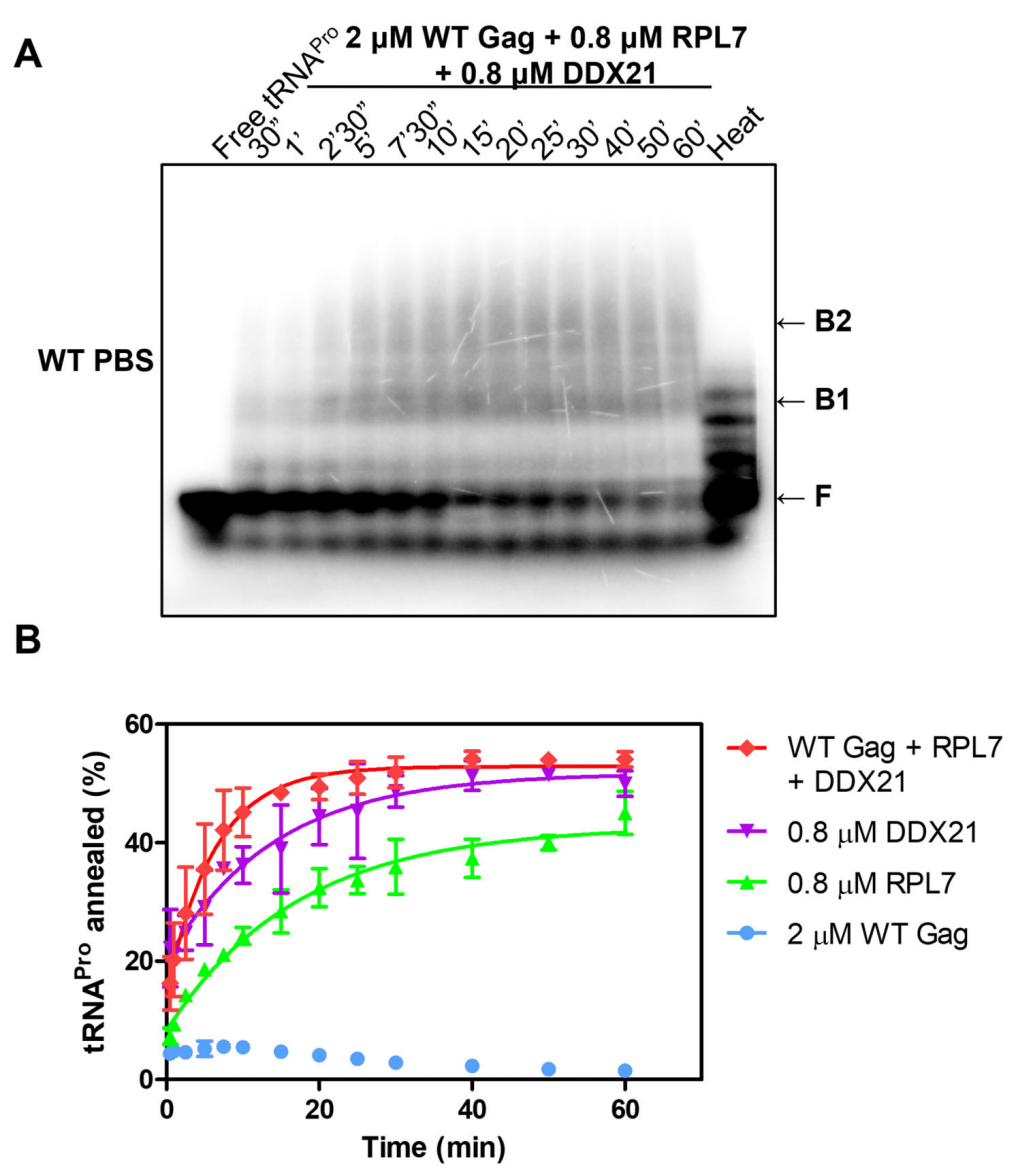
HTLV-1 Gag, RPL7, and DDX21 act synergistically to chaperone tRNA^Pro^ annealing to the HTLV-1 WT PBS. (**A**) Time-course annealing assays using 20 nM 5′ ^32^P-labeled tRNA^Pro^ and 200 nM HTLV-1 WT PBS in the presence of 2 μM HTLV-1 WT Gag, 0.8 μM RPL7, and 0.8 μM DDX21 at 37°C for varying time. F indicates free tRNA^Pro^ and B1 and B2 indicate different tRNA^Pro^-PBS binary complexes. Heat indicates heat annealing, the positive control. Results are representative of three independent experiments. (**B**) Graph for percentages of tRNA^Pro^ annealed to the HTLV-1 WT PBS at different time points in the presence of 2 μM HTLV-1 WT Gag, 0.8 μM RPL7, and 0.8 μM DDX21. Lines represent exponential fits of the data with the standard deviation between three trials indicated.

Time-course annealing assays were also performed in the presence of 0.8 μM RPL7, 0.8 μM DDX21, and 2 μM HTLV-1 WT Gag (Fig. 9A and B). When all three proteins are present, 54% of tRNA^Pro^ is annealed to the WT PBS after 1 h and the scaled annealing rate (*k*′) is 0.086 min^−1^ (Fig. 9A and B; Table 2). This rate is 1.6-fold faster than the combination of RPL7 and DDX21, and 3.3- and 2.4-fold faster than RPL7 or DDX21 alone, respectively. Thus, the maximal synergistic effect on tRNA^Pro^ annealing to the WT PBS is observed with the combination of RPL7, DDX21, and HTLV-1 Gag (Table 2).

**Figure 10.**
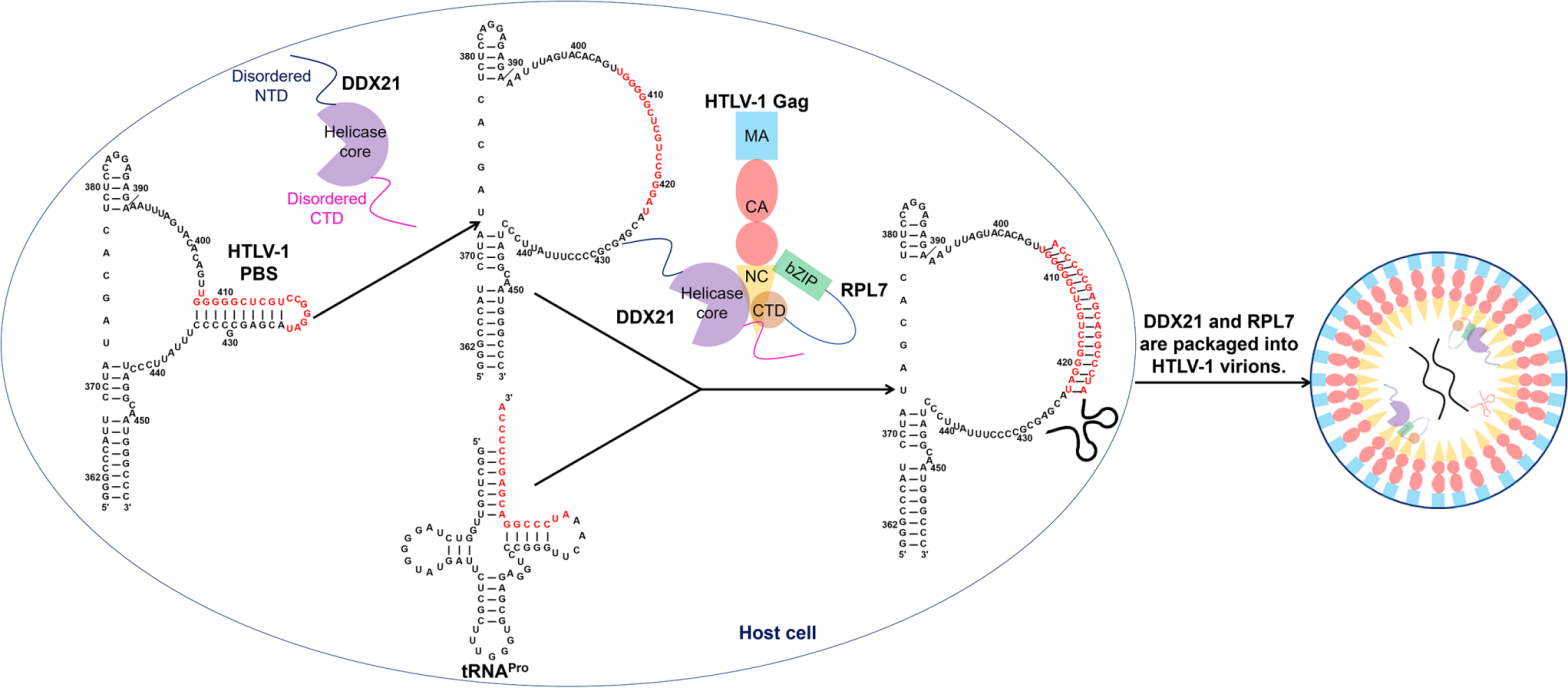
Model for HTLV-1 Gag, human RPL7, and human DDX21 synergistically facilitating tRNA^Pro^ annealing to the HTLV-1 PBS in two steps. In step 1, DDX21 unwinds the stable hairpin in the HTLV-1 PBS region to make the PBS less structured. In step 2, HTLV-1 Gag, RPL7, and DDX21 synergistically facilitate tRNA^Pro^ annealing to the less structured PBS.

Based on these data, our working model for chaperone-facilitated tRNA^Pro^ annealing to the HTLV-1 PBS is shown in Fig. 10. HTLV-1 Gag interacts with RPL7 and DDX21 to form a complex in the host cell and these host factors are packaged into viral particles. tRNA^Pro^ is annealed to the highly-structured HTLV-1 PBS via a two-step mechanism. In the first step, the DDX21 helicase unwinds the PBS stem-loop and in the second step, RPL7, DDX21, and HTLV-1 Gag synergistically anneal tRNA^Pro^ to the more accessible PBS.

## Discussion

Both MA and NC domains of retroviral Gag polyproteins have been reported to possess NA chaperone activity [3, 62]. While HIV-1 Gag is capable of chaperoning tRNA annealing *in vitro* and this activity depends on the NC domain [37], a two-step annealing was proposed in cells. In this mechanism, Gag facilitates partial tRNA^Lys3^ annealing to the PBS in the host cell, and the mature NC domain promotes formation of more complete tRNA^Lys3^-gRNA interaction in virions [63]. HIV-2 Gag has better tRNA^Lys3^ annealing activity *in vitro* than mature MA and NC domains and MA displays stronger annealing activity than NC [64].

In HTLV-1, MA is a more robust NA binding and chaperone protein than NC [16]. In this earlier study, the annealing between HIV-1 TAR RNA and DNA was tested but tRNA annealing to the PBS was not investigated. We now show that neither HTLV-1 Gag, NC, nor MA can chaperone the annealing of tRNA to the highly-structured PBS domain in which the PBS sequence is embedded in a stable hairpin. Even the robust HIV-1 NC protein failed to significantly facilitate this reaction *in vitro* (data not shown).

In this work, we found that HTLV-1 Gag interacts with RPL7 and DDX21 to form a complex (Fig. 3), which increases chaperone activity in a synergistic fashion, with the presence of all three proteins showing more robust activity than any combination of two proteins (Fig. 9 and Supplementary Figs S7–S9). The stoichiometry of the Gag/RPL7/DDX21 complex is unknown. While we showed that HTLV-1 Gag is monomeric in solution (Supplementary Fig. S1B), both RPL7 and DDX21 are capable of forming dimers. RPL7 dimerizes through its N-terminal bZIP domain [52, 65], whereas DDX21 forms dimers through the putative hydrophobic dimerization domain (residues 568–620), which is immediately downstream of the helicase core [60]. Dimer formation of DDX21 is required for its ATP-dependent dsRNA unwinding activity and ATP-independent RNA G-quadruplex resolving activity [60]. Future studies are needed to determine the optimal stoichiometry of the complex for tRNA annealing. In all gel-shift annealing gels (Figs 2, 8, and 9), two conformations of tRNA^Pro^-PBS binary complex are observed. The structural changes of both tRNA^Pro^ and the HTLV-1 5′ UTR after tRNA^Pro^ annealing to the PBS and any additional interactions outside the 18-nt PBS domain remain to be investigated, both in the absence and presence of different combinations of chaperone proteins.

Human RPL7, a ribosomal protein, exhibits tRNA^Pro^ primer annealing activity on its own and both N-terminal bZIP and C-terminal NA-binding domains are involved in this activity (Fig. 8 and Supplementary Fig. S6). Human RPL7 is located on the surface of the large 60S subunit of ribosomes and interacts with 28S rRNA [66]. In addition to serving as building blocks of ribosomes and modulating mRNA translation, numerous ribosomal proteins have been reported to have extra-ribosomal functions, such as RNA chaperone activity [67]. In *E. coli*, approximately a third of 34 large ribosomal proteins tested showed RNA chaperone activity and were proposed to prevent RNAs from being trapped in misfolded forms during translation [68]. In HIV-1-infected cells, RPL7 interacts with the NC domain of HIV-1 Gag and acts synergistically to facilitate annealing of tRNA^Lys3^ to the HIV-1 PBS [21, 22]. Mouse RPL4 increases Gag-Pol readthrough in both Moloney murine leukemia virus and HIV-1, although the mechanism is still unclear [69]. Human RPL4, RPL7A, and RPL6 were top hits in our AP-MS analysis with even greater peptide counts detected than RPL7 or DDX21 (Table 3). Future studies to investigate the potential role of these ribosomal proteins in tRNA primer annealing are warranted.

The human DDX21 helicase also facilitated tRNA^Pro^ annealing to the HTLV-1 PBS on its own and in the absence of ATP (Fig. 8). Of the various proteins tested, DDX21 had the most robust tRNA^Pro^ annealing capability. Helicases that have been reported to be involved in the retroviral lifecycle include RHA (DHX9), DDX1, DDX3, DDX5, DDX17, DDX21, and Moloney leukemia virus 10 homologue (MOV10) [70, 71]. In HIV-1, DDX21 interacts with both the Rev protein and the RRE RNA and facilitates Rev oligomerization on the RRE [28]. Other helicases, such as DDX1, DDX3, DDX5, and DDX17, also facilitate the nuclear export of Rev/RRE-dependent viral unspliced and partially spliced transcripts [28, 61, 72–74]. Although similar functions for these helicases have been reported, knockdown of individual helicase proteins resulted in distinct negative effects on HIV-1 replication, suggesting that they do not play redundant roles in modulating the Rev/RRE pathway [73]. Whether helicases other than DDX21 and RHA [24] facilitate tRNA RT primer annealing in retroviruses remains to be determined. Whether these helicases are also involved in the HTLV-1 Rex/Rex response element-mediated nuclear export of unspliced and partially spliced transcripts is another open question.

Both RPL7 and DDX21 have nuclear localization signals (NLS). RPL7 possesses a tripartite NLS in the bZIP domain and a bipartite NLS in the middle globular domain [75]. Similar NLS features are found in most eukaryotic ribosomal proteins, enabling them to be imported into the nucleus for assembly with rRNA into ribosomes in the nucleolus [76]. In the nucleolus, DDX21 participates in several steps of rRNA biogenesis, including transcription, processing, and small nucleolar ribonucleoprotein (snoRNP)-dependent 2′-O-methylation [77]. Upon virus infection, such as vesicular stomatitis virus and herpes simplex virus 1 (HSV-1), DDX21 translocates from the nucleus to the cytoplasm to sense viral NAs [78]. Both RPL7 and DDX21 can traffic between the nucleus and cytoplasm, and we showed that both are packaged into HTLV-1 virions (Fig. 7). The timing and location of tRNA primer-PBS annealing for any retrovirus remains an open question; the co-packaging of RPL7 and DDX21 into HTLV-1 virions may ensure that tRNA^Pro^ placement onto the PBS is maintained upon transport from the cytoplasm into virions.

In summary, this study identified RPL7 and DDX21 as previously unrecognized host co-factors of HTLV-1 Gag and demonstrated that they function cooperatively with Gag to overcome structural constraints at the highly-structured HTLV-1 PBS, thereby promoting efficient tRNA^Pro^ annealing *in vitro*. Ongoing work is aimed at defining the effect of RPL7 or DDX21 depletion on RT, including primer annealing, elongation, and strand transfer, as well as on viral production and HTLV-1 infectivity. Together, these findings establish primer annealing as a critical regulatory bottleneck in the HTLV-1 replication cycle and reveal host RNA chaperones as key facilitators of this process. Targeting RPL7 or DDX21 RNA remodeling activities, or their interactions with Gag, may represent a novel anti-viral strategy against HTLV-1.

## Supplementary Material

gkag680_Supplemental_File

## Data Availability

All data described are contained within the article. Requests to access the datasets should be directed to K.M.-F. at musier-forsyth.1@osu.edu.

## Notes

Present address: Department of Biochemistry and Molecular Biophysics, Aaron Diamond AIDS Research Center, Columbia University Medical Center, New York, NY 10032, United States
