# Supplementary material for: Human RPL7 and DDX21 function synergistically with HTLV-1 Gag to overcome genomic RNA structural barriers that block tRNAPro primer annealing
Source: Nucleic Acids Res. 2026 Jul 14;54(13):gkag680. doi: 10.1093/nar/gkag680 (PMC13366004; doi:10.1093/nar/gkag680)
Supplement: gkag680_Supplemental_File [file gkag680_supplemental_file.pdf]

SUPPLEMENTARY DATA

Human RPL7 and DDX21 function synergistically with HTLV-1 Gag to overcome genomic RNA structural barriers that block $tRNA^{Pro}$ primer annealing

Yu-Ci Syu[1,2], Zixi Long[1], and Karin Musier-Forsyth[1,]*

[1] Molecular, Cellular, and Developmental Biology Graduate Program, Department of Chemistry and Biochemistry, Center for RNA Biology, and Center for Retrovirus Research, Ohio State University, Columbus, OH 43210, United States

[2] Present Address: Department of Biochemistry and Molecular Biophysics, Aaron Diamond AIDS Research Center, Columbia University Medical Center, New York, NY 10032, United States

* To whom correspondence should be addressed. Email: musier-forsyth.1@osu.edu

MATERIALS AND METHODS

Size-exclusion chromatography with multi-angle laser-light scattering (SEC-MALS)

HTLV-1 WT or ΔC29 Gag proteins (100 µL of 15 µM) were loaded onto a Superdex 200 Increase 10/300 GL size-exclusion column (Cytiva) and eluted with 20 mM Tris-HCl, pH 7.4, 500 mM NaCl, 1 µM $ZnCl_2$, and 5 mM β-mercaptoethanol at a flow rate of 0.5 mL/min. The SEC was controlled by an ÄKTA Pure 25 chromatography system (Cytiva) and MALS analysis was performed by a DAWN HELIOS 8^+ laser detector (Wyatt technology) and an Optilab T-rEX refractometer (Wyatt technology) conjugated to the SEC control system. The light-scattering (LS) intensity and differential refractive index (dRI) of each eluate fraction were measured by the laser detector and the refractometer, respectively. UV, LS, and dRI signals were aligned and the molecular weight of each signal peak was calculated using ASTRA 7.1.4.8 software.

Fluorescence anisotropy (FA) binding assays

RNAs were labeled with fluorescein-5-thiosemicarbazide (FTSC) at the 3'-end and labeling stoichiometry was calculated as described (1). Briefly, the 3'-end of RNA was oxidized by $NaIO_4$ followed by conjugation of FTSC in 1 M NaOAc, pH 5.0. The molar extinction coefficient of FTSC at 495 nm is 85,000 $M^{-1}cm^{-1}$. FA direct-binding assays were used to determine binding affinities as previously described (1). Briefly, 20 nM of 3' FTSC-labeled HTLV-1 PBS RNA and human tRNAs were folded as described in the RNA preparation section and incubated in the dark at room temperature with 0-5 µM HTLV-1 Gag, 0-6.5 µM RPL7, or 0-2 µM DDX21 in 20 mM HEPES, pH 7.5, 50 mM NaCl, and 1 mM $MgCl_2$. FA measurements were performed using a Cytation 5 plate reader (BioTek).

Circular dichroism (CD) spectroscopy

CD spectra were obtained on a Jasco J-815 spectrometer using 2.5 µM WT RPL7 at 25°C in 50 mM sodium phosphate, pH 7.8. CD absorbance values were recorded from 185 to 275 nm with

a data pitch of 0.5 nm at a scanning speed of 100 nm/min. Final CD spectra were plotted against wavelength in nm after subtracting the signal from the buffer blank.

ATPase activity assay

ATPase activity of DDX21 was measured using a malachite green phosphate assay kit as described by the manufacturer (Sigma-Aldrich). Briefly, 250 nM DDX21 was incubated with 0.25 mM ATP and 250 nM folded HTLV-1 PBS RNA at 30 °C in buffer containing 5 mM Tris-HCl, pH 7.8, 125 mM NaCl, 156 nM $MgCl_2$, 0.25 mM TCEP, and 2.5% glycerol. Reaction aliquots of 80 µL were taken at indicated time points and mixed with 20 µL of working reagent from the assay kit (100 volumes of reagent A and 1 volume of reagent B). For both the reactions and phosphate standards, the absorption at 620 nm was monitored using a Cytation 5 plate reader (BioTek). The amount of released inorganic phosphate in the reactions was determined using a standard curve generated from the phosphate standards. The ATP hydrolysis rate was determined from the slope of the linear increase in phosphate production over time and normalized to the DDX21 protein concentration.

FIGURES AND TABLES

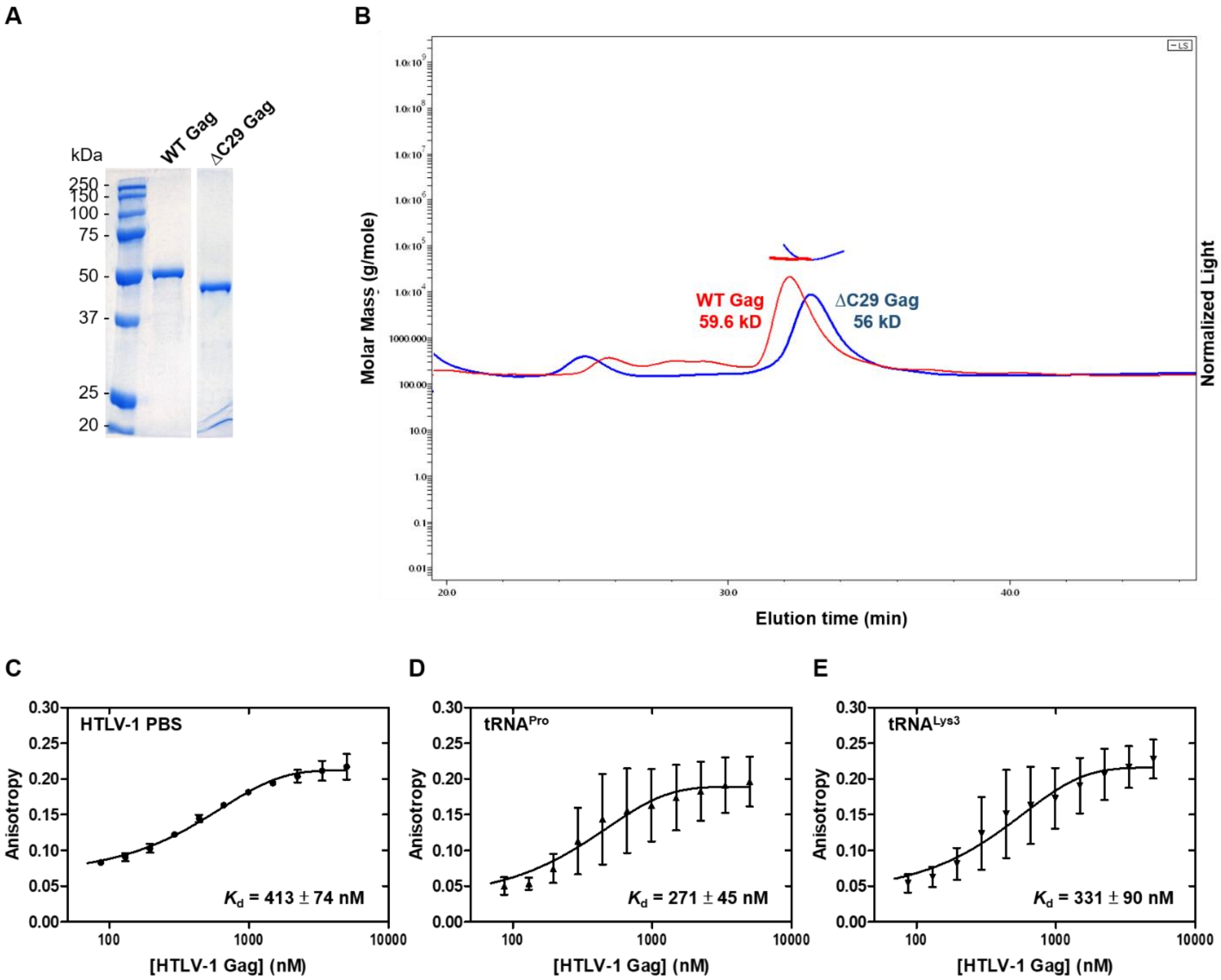

Figure S1. Recombinant HTLV-1 Gag proteins purified from *E. coli* and characterized by size-exclusion chromatography with multi-angle laser-light scattering (SEC-MALS) analysis and fluorescence anisotropy (FA) RNA binding assays. (A) Recombinant HTLV-1 WT and ΔC29 Gag proteins (1 µg) purified from *E. coli* were run on an SDS-polyacrylamide (10%) gel and stained with Coomassie blue. Only one major band that migrated close to the expected molecular weight was observed for each protein. (B) SEC-MALS chromatogram of purified HTLV-1 Gag proteins. Normalized light scattering peaks for HTLV-1 WT (red) and ΔC29 (blue) Gag are shown. The molar mass range for each peak was calculated and indicated by thick lines. (C-E) FA binding curves showing recombinant HTLV-1 Gag protein

(0-5 μM) binding to 20 nM 3' FTSC-labeled HTLV-1 PBS RNA (C), $tRNA^{Pro}$ (D), and $tRNA^{Lys3}$ (E) in 20 mM HEPES, pH 7.5, 50 mM NaCl, and 1 mM $MgCl_2$. K_d values are the average of two independent experiments.

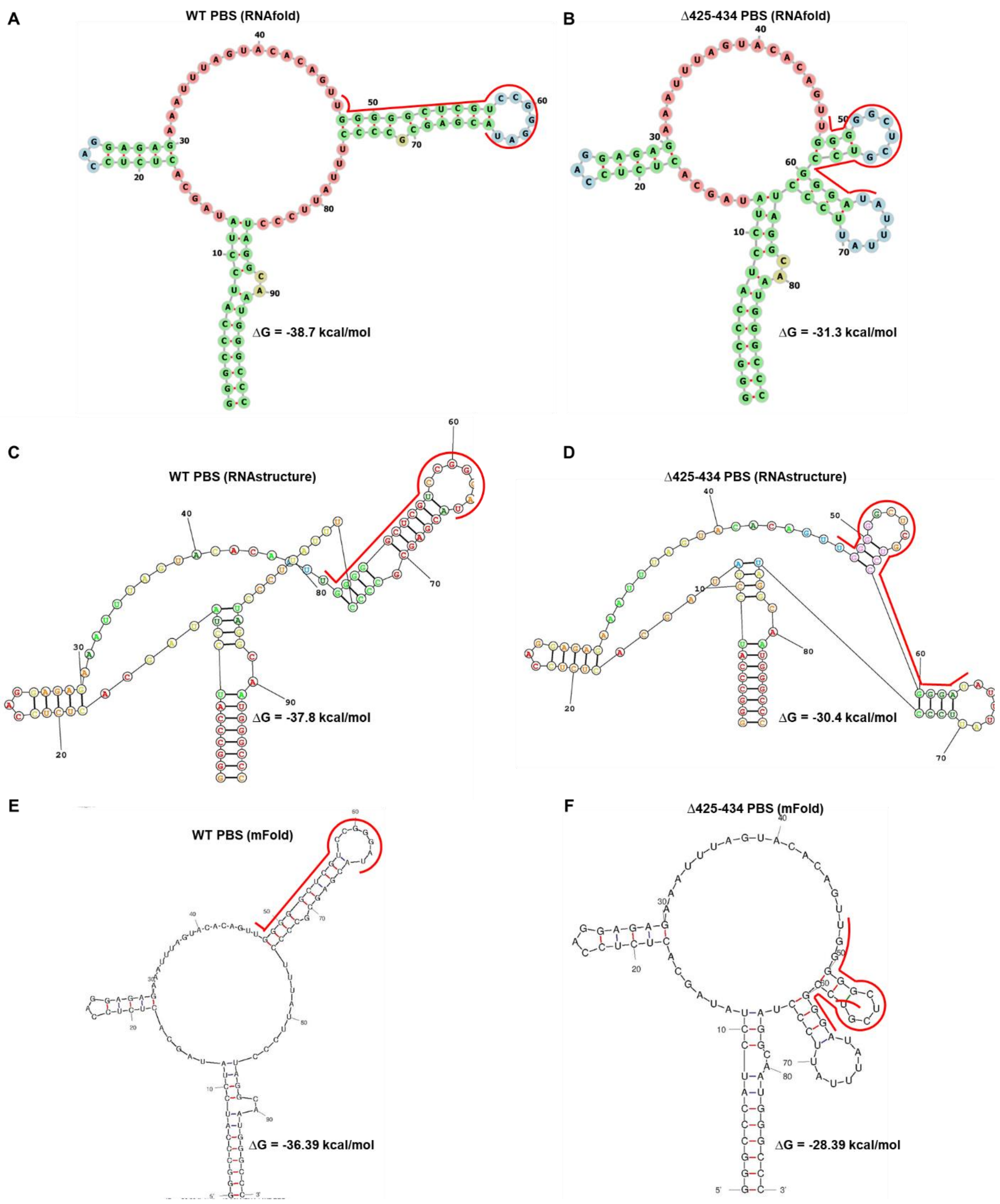

Figure S2. Secondary structures of HTLV-1 PBS RNAs predicted by RNAfold (2), RNAstructure (3), and mFold (4). RNA folding programs RNAfold (A and B), RNAstructure (C and D), and mFold (E and F), were used to predict secondary structures of WT PBS RNA (A,

C, and E) and Δ425-434 PBS RNA (B, D, and F). The PBS regions are highlighted in red, and ΔG values are indicated for each prediction.

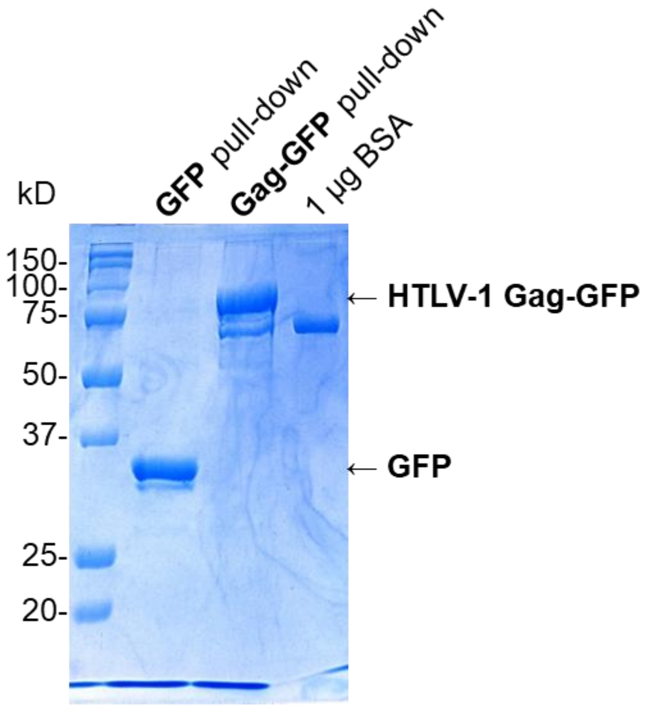

Figure S3. Affinity tagging/purification-mass spectrometry (AP-MS) used to identify interacting partners of HTLV-1 Gag. Prior to AP-MS analysis, GFP or HTLV-1 Gag-GFP was pulled-down from HEK293T cell lysate by streptactin, run on an SDS-polyacrylamide gel (10%), and stained with Coomassie blue.

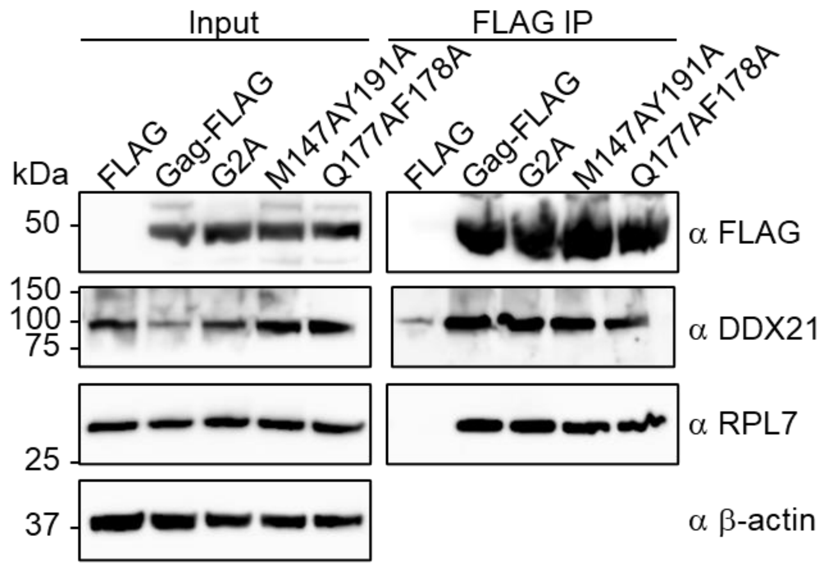

Figure S4. Western blot showing that interactions between HTLV-1 Gag and RPL7 or DDX21 are Gag-myristoylation- and Gag-oligomerization-independent. FLAG alone and WT, G2A, M147AY191A, and Q177AF178A Gag-FLAG in HEK293T cell lysate were immunoprecipitated and immunoblotted using an anti-FLAG antibody. Co-immunoprecipitated proteins were immunoblotted using anti-DDX21 and anti-RPL7 antibodies. β-actin was used as a loading control. Results are representative of three independent experiments.

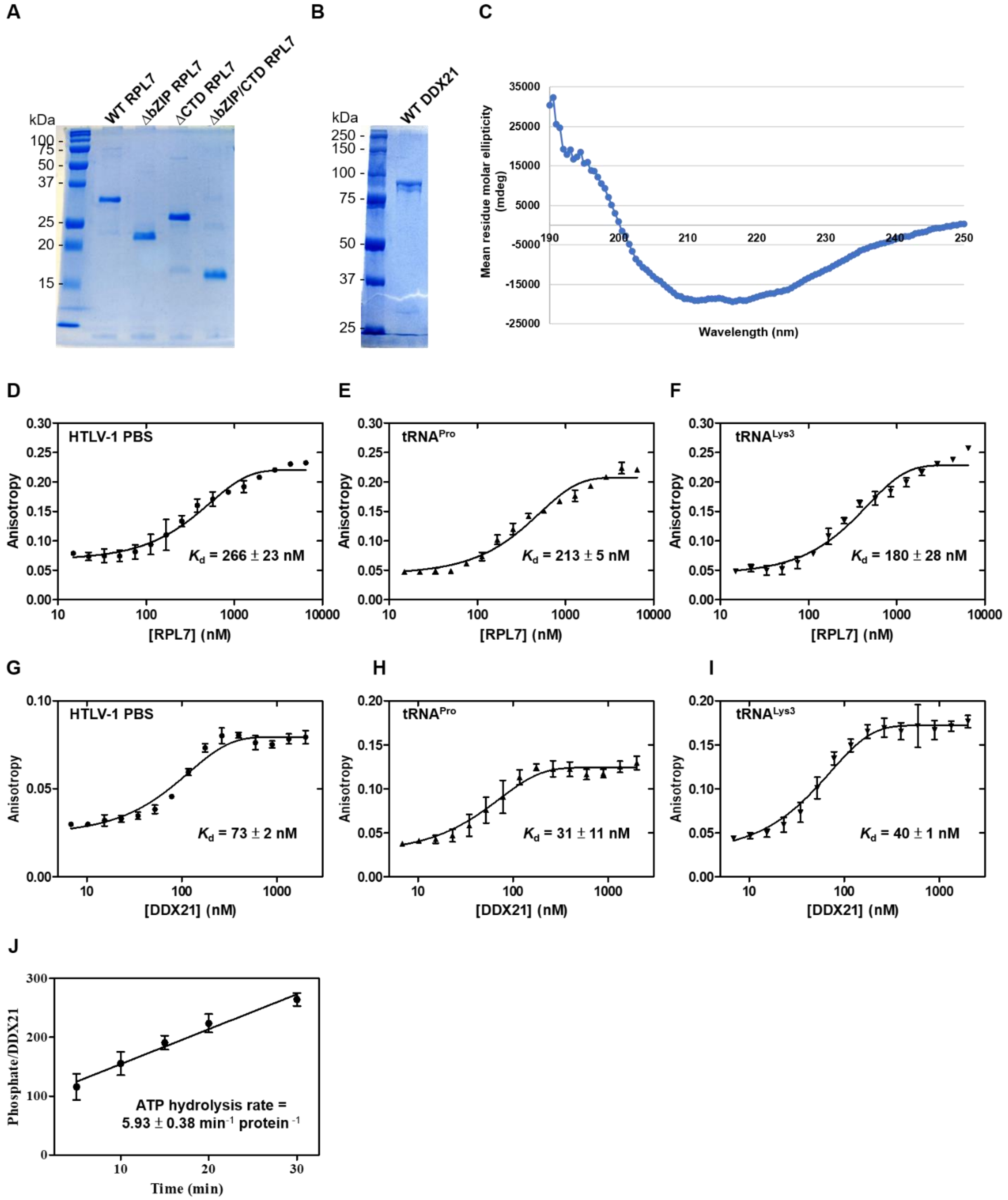

Figure S5. Characterization of the purity, folding, and function of RPL7 and DDX21 proteins. (A) Coomassie blue-stained SDS-polyacrylamide (12%) gel showing recombinant human WT, ΔbZIP, ΔCTD, and ΔbZIP/CTD RPL7 proteins (1 μg) purified from *E. coli*. (B)

Coomassie blue-stained SDS-polyacrylamide (8%) gel showing recombinant human WT DDX21 protein (1 µg) purified from *E. coli.* (C) Circular dichroism (CD) spectrum of WT RPL7. (D-I) FA assays showing recombinant RPL7 (0-6.5 µM, D-F) and DDX21 (0-2 µM, G-I) binding to 20 nM 3' FTSC-labeled HTLV-1 PBS RNA (D and G), $tRNA^{Pro}$ (E and H), and $tRNA^{Lys3}$ (F and I) in 20 mM HEPES, pH 7.5, 50 mM NaCl, and 1 mM $MgCl_2$. K_d values are the average of two and three independent experiments for RPL7 and DDX21, respectively. (J) DDX21 ATPase activity measured using malachite green assay. Released inorganic phosphate is plotted as a function of time and the ATP hydrolysis rate was determined from the slope of the line. The rate represents the average of three independent experiments normalized to protein concentration.

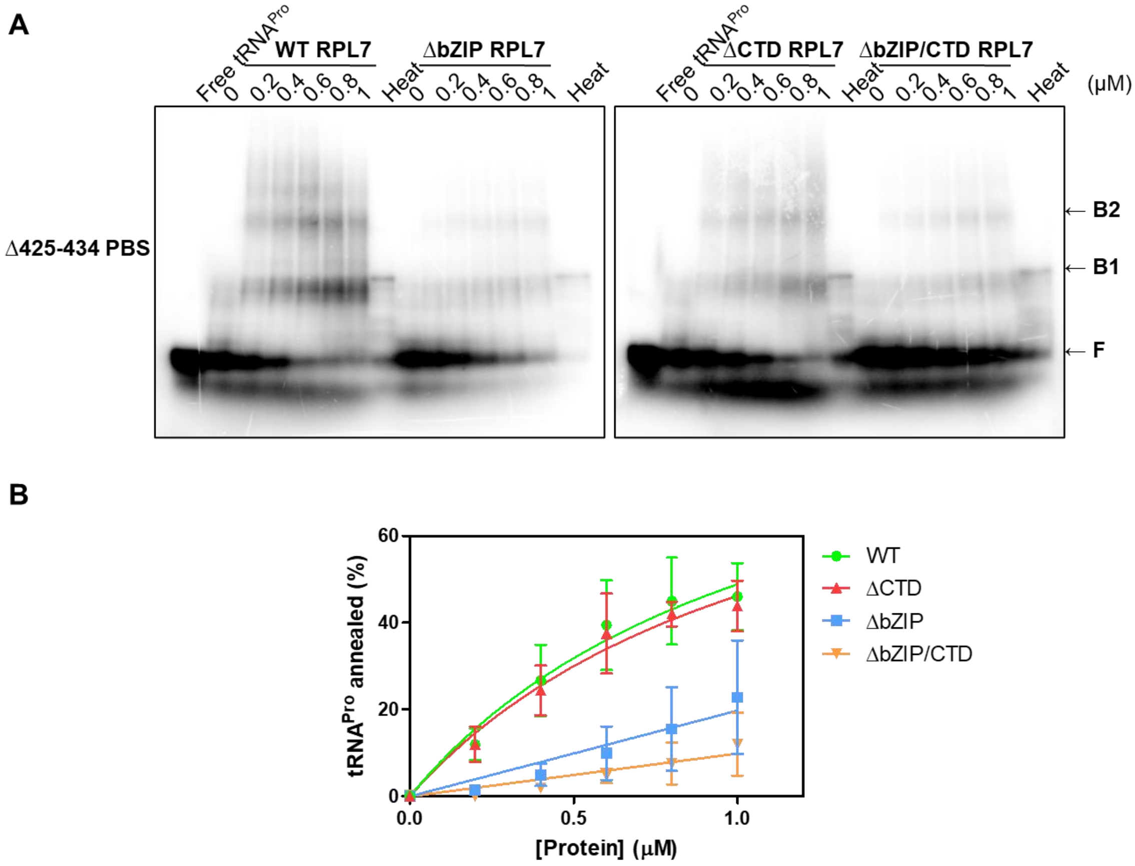

Figure S6. Gel-shift assays showing that both bZIP and CTD of RPL7 are involved in chaperoning tRNA^{Pro} annealing to the HTLV-1 PBS. (A) Concentration-dependence annealing assays were performed using 20 nM 5' ^{32}P-labeled tRNA^{Pro} and 200 nM HTLV-1 Δ425-434 PBS in the presence of varying concentrations of WT, ΔbZIP, ΔCTD, or ΔbZIP/CTD RPL7 at 37°C for 1 h. F indicates free tRNA^{Pro}, and B1 and B2 indicate different conformations of tRNA^{Pro}-PBS binary complexes. Heat indicates heat annealing, the positive control. Results are representative of three independent experiments. (B) Graph showing percentages of tRNA^{Pro} annealed to the Δ425-434 PBS in varying concentrations of WT, ΔbZIP, ΔCTD, or ΔbZIP/CTD RPL7. Lines represent exponential fits of the data with the standard deviation between three trials indicated.

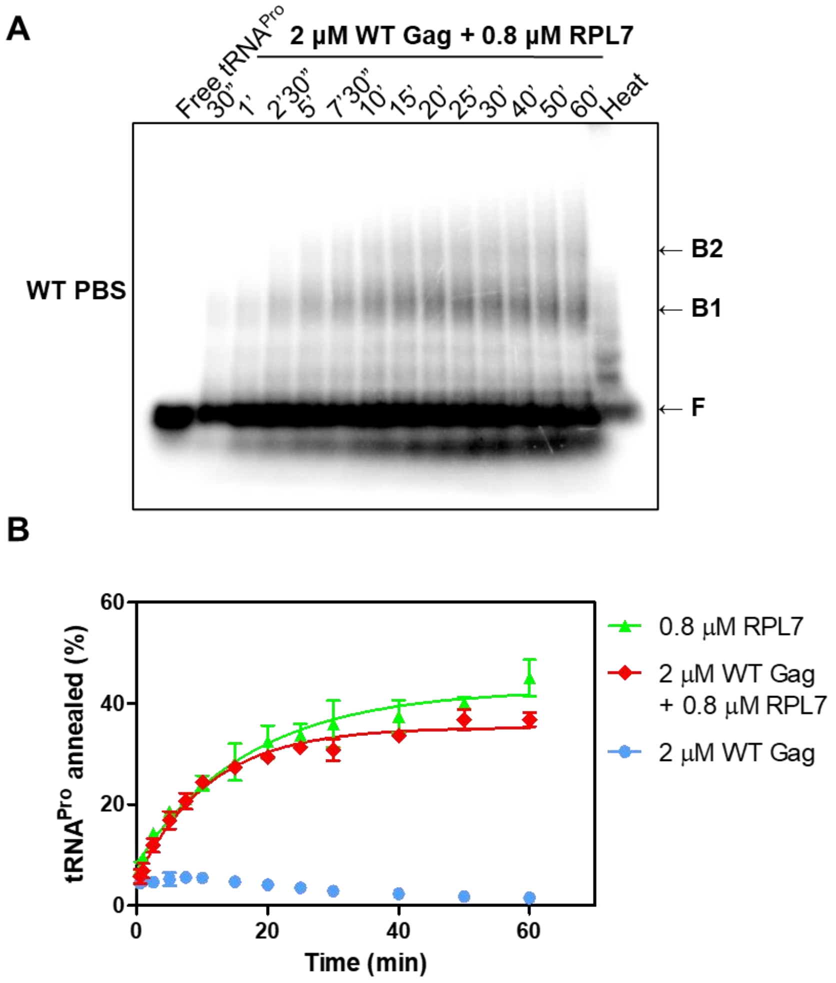

Figure S7. Gel-shift assays showing that in the presence of both RPL7 and HTLV-1 Gag, no synergistic annealing of tRNA^{Pro} to the HTLV-1 PBS is observed. (A) Time-course annealing assays were performed using 20 nM 5' ^{32}P-labeled tRNA^{Pro} and 200 nM HTLV-1 WT PBS in the presence of 0.8 μM RPL7 and 2 μM HTLV-1 WT Gag at 37°C. F indicates free tRNA^{Pro}, and B1 and B2 indicate different tRNA^{Pro}-PBS binary complexes. Heat indicates heat annealing, the positive control. Results are representative of three independent experiments. (B) Graph showing percentages of tRNA^{Pro} annealed to the HTLV-1 WT PBS at different time points in the presence of 0.8 μM RPL7 and 2 μM HTLV-1 WT Gag. Lines represent exponential fits of the data with the standard deviation between three trials indicated.

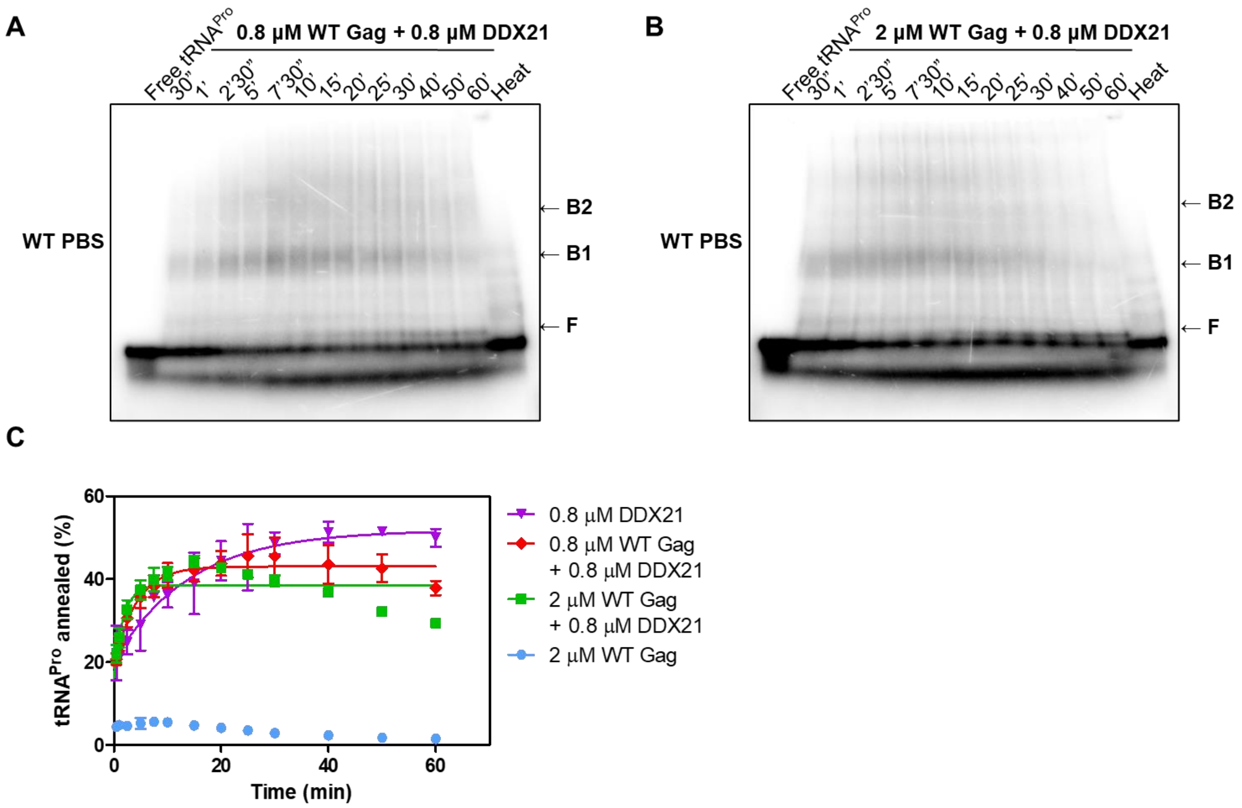

Figure S8. Gel-shift assays showing that in the presence of both DDX21 and HTLV-1 WT Gag, no synergistic tRNA^{Pro} annealing to the HTLV-1 PBS is observed. (A and B) Time-course annealing assays were performed using 20 nM 5' ^{32}P-labeled tRNA^{Pro} and 200 nM HTLV-1 WT PBS in the presence of 0.8 µM DDX21 and 0.8 µM (A) or 2 µM (B) HTLV-1 WT Gag at 37°C. F indicates free tRNA^{Pro}, and B1 and B2 indicate different conformations of tRNA^{Pro}-PBS binary complexes. Heat indicates heat annealing, the positive control. Results are representative of three independent experiments. (C) Graph showing percentages of tRNA^{Pro} annealed to the HTLV-1 WT PBS at different time points in the presence of 0.8 µM DDX21 and 0.8 µM or 2 µM HTLV-1 WT Gag. Lines represent exponential fits of the data with the standard deviation between three trials indicated.

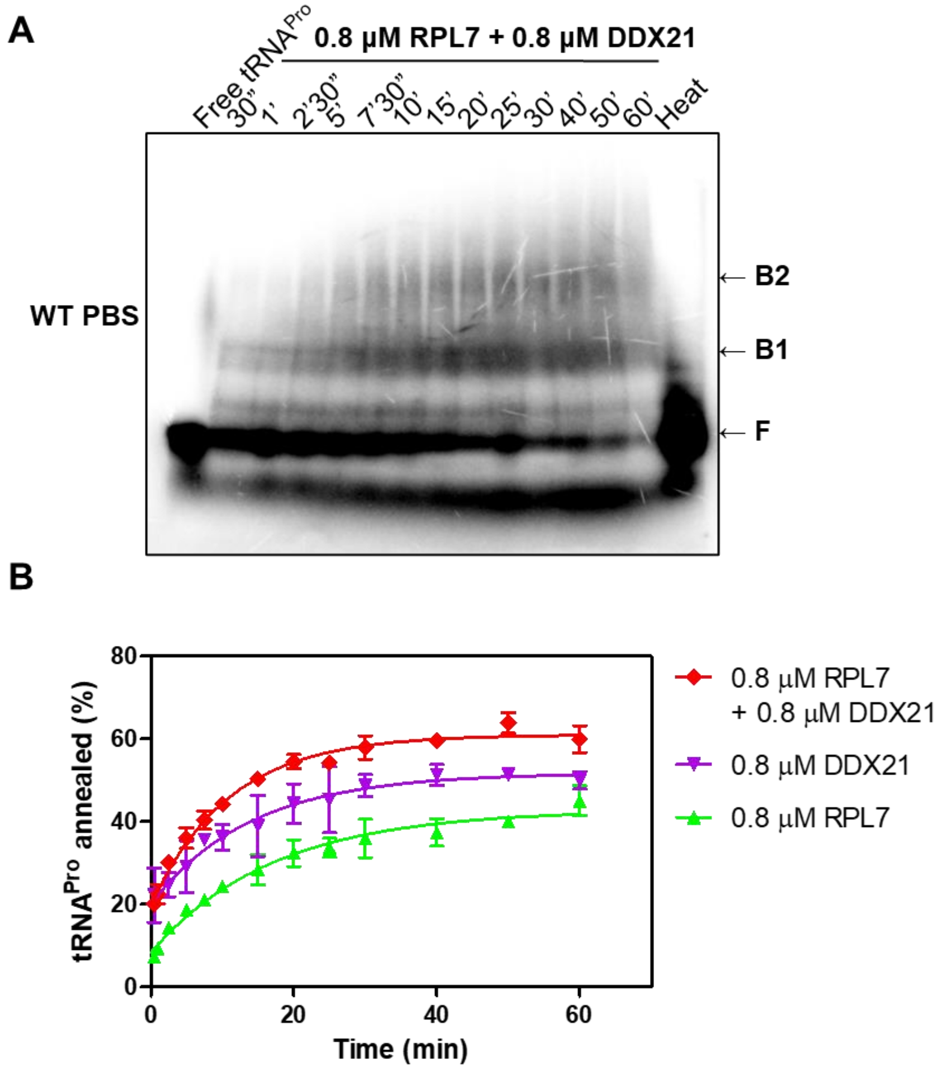

Figure S9. Gel-shift assays showing that RPL7 and DDX21 act synergistically to chaperone $tRNA^{Pro}$ annealing to the HTLV-1 PBS. (A) Time-course annealing assays performed using 20 nM 5' ^{32}P-labeled $tRNA^{Pro}$ and 200 nM HTLV-1 WT PBS in the presence of 0.8 μM RPL7 and 0.8 μM DDX21 at 37°C. F indicates free $tRNA^{Pro}$, and B1 and B2 indicate different $tRNA^{Pro}$-PBS binary complexes. Heat indicates heat annealing, the positive control. Results are representative of three independent experiments. (B) Graph showing percentages of $tRNA^{Pro}$ annealed to the HTLV-1 WT PBS at different time points in the presence of 0.8 μM RPL7 and 0.8 μM DDX21. Lines represent exponential fits of the data with the standard deviation between three trials indicated.

Table S1. List of primer sets used for generating pET3xc HTLV-1 Gag, pUCOgs HTLV-1 Gag-GFP, pUCOgs HTLV-1 Gag-FLAG, pPB RPL7, pCMV3 HA-RPL7, pLV CMV mCherry-DDX21-V5, and pUC19 HTLV-1 PBS mutant constructs.

Mutant		Primer sequence
**pET3xc HTLV-1 Gag**	**ΔC29**	Forward primer: 5'-GAA AAC CTT TAC TTC CAG GGC GAT TAC G-3'<br>Reverse primer:5'- GAA GTA AAG GTT TTC AAT GGT TGG CTT CAG-3'
**pUCOgs HTLV-1 Gag-GFP**	**ΔMA**	Forward primer: 5'-CAA GCT TGC CAC CAT GCC TGT CAT GCA CC-3'<br>Reverse primer: 5'-CAT GGT GGC AAG CTT GAG CTC GAG ATC TG-3'
	**ΔCA**	Forward primer: 5'-GTG GTG CAG CCT AAA AAG CCC CCC CCG AAT C-3'<br>Reverse primer: 5'-TAG GCT GCA CCA CCA GCA CTT GGG GGG CGG-3'
	**ΔNC**	Forward primer: 5'-CAA GAC CAA GGT GCT GGG ATC CCT TGA AGT G-3'<br>Reverse primer: 5'-CAG CAC CTT GGT CTT GTC CTT GGG GGT CCA G-3'
	**ΔZF1**	Forward primer: 5'-CAC AGC CCA GAC CTC CCC CAG GAC CAT GC-3'<br>Reverse primer: 5'-GAG GTC TGG GCT GTG TGG GCT GAT TCG GGG-3'
	**ΔZF2**	Forward primer: 5'-CCA AGA CTG AAG CCA ACC ATT CCT GAG CC-3'<br>Reverse primer: 5'-GGC TTC AGT CTT GGT GGT CCT GGG GGA GGT C-3'
	**ΔNC CTD (ΔC29)**	Forward primer: 5'-CTG AAG CCA ACC ATT GGA TCC CTT GAA GTG-3'<br>Reverse primer: 5'-AAT GGT TGG CTT CAG TCT TGG GCA GTC GC-3'
**pUCOgs HTLV-1 Gag-GFP or Gag-FLAG**	**G2A**	Forward primer: 5'-ATG GCC CGC ATT TTC AGC CGC TCC GCT TC-3'<br>Reverse primer: 5'-GAA AAT GCG GGC CAT GGT GGC AAG CTT GAG-3'
	**M147A**	Forward primer: 5'-GGC AGG CCA AGG ATC TCC AGG CTA TTA AGC-3'<br>Reverse primer: 5'-GAT CCT TGG CCT GCC AAG GGC GGT GGT TG-3'
	**F177AQ178A**	Forward primer: 5'-CGT GCA AGC TGC TGA TCC AAC CGC TAA GG-3'<br>Reverse primer: 5'-CAG CAG CTT GCA CGG CCA GCC TAA TTG TC-3'
	**Y191A**	Forward primer: 5'-CAG GCC CTT TGC TCC AGC CTC GTC GCC AGC-3'

		Reverse primer: 5'-GGA GCA AAG GGC CTG CAG CAG GTC CTG GAG-3'
**pPB RPL7**	**ΔbZIP (ΔN77)**	Forward primer: 5'-GCT GGC AAC TTC TAT GTA CCT GCA GAA CC-3'<br>Reverse primer: 5'-CAT AGA AGT TGC CAG CGC CAA CTT TTT TG-3'
	**ΔCTD (ΔC51)**	Forward primer: 5'-GAG ATC TAT ACT GTT TGC CCA ACT TTC TTG-3'<br>Reverse primer: 5'-CAG TAT AGA TCT CAT GAA TCA AAT CCT CC-3'
**pCMV3 HA-RPL7**	**ΔbZIP (ΔN77)**	Forward primer: 5'-GCT GGC AAC TTC TAT GTA CCT GCA GAA CC-3'<br>Reverse primer: 5'-GAA GTT GCC AGC GCT ACC GCC TCC ACC-3'
	**bZIP (N77)-(GGGGS)$_3$-CTD (C51)**	Forward primer: 5'-GGT GGA GGA GGT AGC GGT GGT GGA GGA TCT GGA AAA CGC TTC AAA G-3'<br>Reverse primer: 5'-GCT ACC TCC TCC ACC AGA TCC ACC ACC TCC TTT TCT TGC CAT CCT C-3'
	**ΔCTD (ΔC51)**	Forward primer: 5'-GAG ATC TAT ACT GTT TGA ATC TAG AGC GGC-3'<br>Reverse primer: 5'-CAG TAT AGA TCT CAT GAA TCA AAT CCT CC-3'
	**bZIP (N77)**	Forward primer: 5'-GAG GAT GGC AAG AAA ATG AAT CTA GAG CGG C-3'<br>Reverse primer: 5'-CTT GCC ATC CTC GCC ATT CGA ATT TCA GTT C-3'
	**CTD (C51)**	Forward primer: 5'-GGA AAA CGC TTC AAA GAG GCA AAT AAC-3'<br>Reverse primer:5'-GAA GCG TTT TCC GCT ACC GCC TCC ACC-3'
**pLV CMV mCherry-DDX21-V5**	**ΔNTD**	Forward primer: 5'- GGC GCT TTC TCT AAT TTT CCC ATA TCT G-3'<br>Reverse primer: 5'- AGA GAA AGC GCC CAT GCT AGC TGA TCC G-3'
	**ΔHelicase N**	Forward primer: 5'- GTG GAA CAA AAA GAA GTG GAG CAT CTG GC-3'<br>Reverse primer: 5'- CTT TTT GTT CCA CAG GTA TTT C-3'
	**ΔHelicase C**	Forward primer: 5'- GAA AAC GGC AAT AAC TGC AAC AGA AAT AAT AAA AG-3'<br>Reverse primer: 5'- GTT ATT GCC GTT TTC TGA GTC-3'
	**ΔCTD**	Forward primer: 5'- GAA TAG GTG TTC CTT CTG CCT CGA GTC TAG AG-3'<br>Reverse primer: 5'- GAA GGA ACA CCT ATT CGT TTG-3'
	**ΔHelicase core**	Forward primer: 5'- GTG GAA CAA AAA GAA GCA ACA GAA ATA ATA AAA G-3'<br>Reverse primer: 5'- CTT TTT GTT CCA CAG GTA TTT C-3'
**pUC19 HTLV-1 PBS**	**Δ425-434**	Tailed forward primer: 5'-GGC TCG TCC GGG ATA TTT ATT CCC TAG GCA ATG GG-3'<br>Forward primer: 5'-TTT ATT CCC TAG GCA ATG GG-3'<br>Tailed reverse primer: 5'-TAT CCC GGA CGA GCC CCC AAC TGT GTA CTA AAT TTC TC-3'<br>Reverse primer: 5'-CCC AAC TGT GTA CTA AAT TTC TC-3'

Table S2. List of RNA sequences used in this study.

RNA	Length (nt)	Sequence
HTLV-1 WT PBS[α]	98	5'-*GG*G CCC AUC CUA UAG CAC UCU CCA GGA GAG AAA UUU AGU ACA CAG UUG GGG GCU CGU CCG GGA UAC GAG CGC CCC UUU AUU CCC UAG GCA AUG GGC C*C*-3'
HTLV-1 Δ425-434 PBS[α]	88	5'-*GG*G CCC AUC CUA UAG CAC UCU CCA GGA GAG AAA UUU AGU ACA CAG UUG GGG GCU CGU CCG GGA UAU UUA UUC CCU AGG CAA UGG GCC *C*-3'
$tRNA^{Pro}_{UGG}$	75	5'-GGC UCG UUG GUC UAG GGG UAU GAU UCU CGC UUU GGG UGC GAG AGG UCC CGG GUU CAA AUC CCG GAC GAG CCC CCA-3'

[α] The sequences underlined are the HTLV-1 18-nt PBS. Italicized 5' 2 G's are not encoded by HTLV-1 and were added for efficient initiation of *in vitro* transcription by T7 RNA polymerase.

Table S3. List of all AP-MS protein hits.

Gene symbol	Protein name	Total Spectrum Count	
		Gag-GFP	GFP
RPN1	Dolichyl-diphosphooligosaccharide--protein glycosyltransferase subunit 1	50	4
RPL4	60S ribosomal protein L4	48	1
ABCD3	ATP-binding cassette sub-family D member 3	37	4
RPL7A	60S ribosomal protein L7a	34	2
RPL6	60S ribosomal protein L6	31	0
**RPL7**	**60S ribosomal protein L7**	25	0
RPS3A	40S ribosomal protein S3a	23	3
**DDX21**	**Nucleolar RNA helicase 2**	22	0
RPL18	60S ribosomal protein L18	20	0
DDOST	Dolichyl-diphosphooligosaccharide--protein glycosyltransferase 48 kDa subunit	20	1
RPLP2	60S acidic ribosomal protein P2	20	3
RPL12	60S ribosomal protein L12	19	0
RPS8	40S ribosomal protein S8	19	0
ATG2A	Autophagy-related protein 2 homolog A	19	0
RPN2	Dolichyl-diphosphooligosaccharide--protein glycosyltransferase subunit 2	18	0
MCU	Calcium uniporter protein, mitochondrial	18	1
LBR	Lamin-B receptor	18	3
NMT1	Glycylpeptide N-tetradecanoyltransferase 1	17	0
RPL10A	60S ribosomal protein L10a	17	1
RPL15	60S ribosomal protein L15	16	0
GNL3	Guanine nucleotide-binding protein-like 3	16	1
RPS6	40S ribosomal protein S6	16	2
MYBBP1A	Myb-binding protein 1A	15	0
RPL23A	60S ribosomal protein L23a	14	0
RPL8	60S ribosomal protein L8	14	0
DHX9	ATP-dependent RNA helicase A	14	3
RPL27	60S ribosomal protein L27	13	0
TMEM43	Transmembrane protein 43	13	1
LMAN2	Vesicular integral-membrane protein VIP36	12	0
ALG8	Probable dolichyl pyrophosphate Glc1Man9GlcNAc2 alpha-1,3-glucosyltransferase	12	0
DGAT1	Diacylglycerol O-acyltransferase 1	11	0
NOP2	Probable 28S rRNA (cytosine(4447)-C(5))-methyltransferase	11	0
NOM1	Nucleolar MIF4G domain-containing protein 1	11	0
GPC4	Glypican-4	10	0
HSPA4	Heat shock 70 kDa protein 4	10	0
BCAP31	B-cell receptor-associated protein 31	10	0

ABCB10	ATP-binding cassette sub-family B member 10, mitochondrial	10	0
NOP56	Nucleolar protein 56	10	1
RBM4	RNA-binding protein 4	9	0
SCAMP3	Secretory carrier-associated membrane protein 3	9	0
PEX10	Peroxisome biogenesis factor 10	9	0
SON	SON DNA binding protein	9	0
PTDSS1	Phosphatidylserine synthase 1	9	0
PIGK	GPI-anchor transamidase	9	0
RRBP1	Ribosome-binding protein 1	9	0
SEC61A1	Protein transport protein Sec61 subunit alpha isoform 1	9	1
SEC61A2	Protein transport protein Sec61 subunit alpha isoform 2	8	0
RBM4B	RNA-binding protein 4B	8	0
RPLP0	60S acidic ribosomal protein P0	8	0
MLEC	Malectin	8	0
STEAP3	Metalloreductase STEAP3	8	0
DHX30	Putative ATP-dependent RNA helicase DHX30	8	0
RPL34	60S ribosomal protein L34	8	1
SAAL1	Synoviocyte proliferation-associated in collagen-induced arthritis protein 1	8	1
TUBGCP2	Gamma-tubulin complex component 2	8	1
YBX1	Nuclease-sensitive element-binding protein 1	7	0
CAND2	Cullin-associated NEDD8-dissociated protein 2	7	0
CYB5R3	NADH-cytochrome b5 reductase 3	7	0
RPL35A	60S ribosomal protein L35a	7	0
SOAT1	Sterol O-acyltransferase 1	7	0
RPL18A	60S ribosomal protein L18a	7	0
EMC1	ER membrane protein complex subunit 1	7	0

References

1. Rye-McCurdy, T., Rouzina, I. and Musier-Forsyth, K. (2015) Fluorescence anisotropy-based salt-titration approach to characterize protein-nucleic acid interactions. *Methods Mol Biol*, **1259**, 385-402.
2. Lorenz, R., Bernhart, S.H., Honer Zu Siederdissen, C., Tafer, H., Flamm, C., Stadler, P.F. and Hofacker, I.L. (2011) ViennaRNA Package 2.0. *Algorithms Mol Biol*, **6**, 26.
3. Reuter, J.S. and Mathews, D.H. (2010) RNAstructure: software for RNA secondary structure prediction and analysis. *BMC Bioinformatics*, **11**, 129.
4. Zuker, M. (2003) Mfold web server for nucleic acid folding and hybridization prediction. *Nucleic Acids Res*, **31**, 3406-3415.
